# Long-Term Effect of Home Blood Pressure Self-Monitoring Plus Medication Self-Titration for Patients With Hypertension: A Secondary Analysis of the ADAMPA Randomized Clinical Trial

**DOI:** 10.1001/jamanetworkopen.2024.10063

**Published:** 2024-05-10

**Authors:** Patricia Martínez-Ibáñez, Irene Marco-Moreno, Aníbal García-Sempere, Salvador Peiró, Lucia Martínez-Ibáñez, Ignacio Barreira-Franch, Laura Bellot-Pujalte, Eugenia Avelino-Hidalgo, Marina Escrig-Veses, María Bóveda-García, Mercedes Calleja-del-Ser, Celia Robles-Cabaniñas, Isabel Hurtado, Clara L. Rodríguez-Bernal, Margarita Giménez-Loreiro, Gabriel Sanfélix-Gimeno, José Sanfélix-Genovés

**Affiliations:** 1 Health Services Research & Pharmacoepidemiology Unit, Fundació per al Foment de la Investigació Sanitària i Biomèdica de la Comunitat Valenciana (Fisabio), Valencia, Spain; 2 Network for Research on Chronicity, Primary Care, and Health Promotion (RICAPPS), Spain; 3 INCLIVA Health Research Institute, Valencia, Spain

## Abstract

**Importance:**

Patient empowerment through pharmacologic self-management is a common strategy for some chronic diseases such as diabetes, but it is rarely used for controlling blood pressure (BP). Several trials have shown its potential for reducing BP in the short term, but evidence in the longer term is scarce.

**Objective:**

To evaluate the longer-term effectiveness of BP self-monitoring plus self-titration of antihypertensive medication vs usual care for patients with poorly controlled hypertension, with passive follow-up and primary-care nursing involvement.

**Design, Setting, and Participants:**

The ADAMPA (Impact of Self-Monitoring of Blood Pressure and Self-Titration of Medication in the Control of Hypertension) study was a randomized, unblinded clinical trial with 2 parallel arms conducted in Valencia, Spain. Included participants were patients 40 years or older, with systolic BP (SBP) over 145 mm Hg and/or diastolic BP (DBP) over 90 mm Hg, recruited from July 21, 2017, to June 30, 2018 (study completion, August 25, 2020). Statistical analysis was conducted on an intention-to-treat basis from August 2022 to February 2024.

**Interventions:**

Participants were randomized 1:1 to usual care vs an individualized, prearranged plan based on BP self-monitoring plus medication self-titration.

**Main Outcomes and Measures:**

The main outome was the adjusted mean difference (AMD) in SBP between groups at 24 months of follow-up. Secondary outcomes were the AMD in DBP between groups at 24 months of follow-up, proportion of patients reaching the BP target (SBP <140 mm Hg and DBP <90 mm Hg), change in behaviors, quality of life, health service use, and adverse events.

**Results:**

Among 312 patients included in main trial, data on BP measurements at 24 months were available for 219 patients (111 in the intervention group and 108 in the control group). The mean (SD) age was 64.3 (10.1) years, and 120 patients (54.8%) were female; the mean (SD) SBP was 155.6 (13.1) mm Hg, and the mean (SD) diastolic BP was 90.8 (7.7) mm Hg. The median follow-up was 23.8 months (IQR, 19.8-24.5 months). The AMD in SBP at the end of follow-up was −3.4 mm Hg (95% CI, −4.7 to −2.1 mm Hg; *P* < .001), and the AMD in DBP was −2.5 mm Hg (95% CI, −3.5 to −1.6 mm Hg; *P* < .001). Subgroup analysis for the main outcome showed consistent results. Sensitivity analyses confirmed the robustness of the main findings. No differences were observed between groups in behaviors, quality of life, use of health services, or adverse events.

**Conclusions and Relevance:**

In this secondary analysis of a randomized clinical trial, BP self-monitoring plus self-titration of antihypertensive medication based on an individualized prearranged plan used in primary care reduced BP in the longer term with passive follow-up compared with usual care, without increasing health care use or adverse events. These results suggest that simple, inexpensive, and easy-to-implement self-management interventions have the potential to improve the long-term control of hypertension in routine clinical practice.

**Trial Registration:**

ClinicalTrials.gov Identifier: NCT03242785

## Key Points

**Question:**

Does a self-management intervention based on home blood pressure monitoring and self-titration of antihypertensive medication allow for better control of blood pressure compared with usual care at 24 months?

**Findings:**

In this prespecified secondary analysis of a randomized clinical trial of 219 patients with uncontrolled hypertension, a self-management intervention including home blood pressure monitoring and self-titration of antihypertensive medication resulted in a statistically significant reduction in systolic blood pressure (adjusted mean difference, −3.4 mm Hg) and diastolic blood pressure (adjusted mean difference, −2.5 mm Hg) at 24 months, with no increase in the use of health care resources or adverse events compared with usual care.

**Meaning:**

Hypertension self-management strategies for patients in primary care may be effective to control blood pressure in the longer term.

## Teaser

This prespecified secondary analysis of the ADAMPA randomized clinical trial evaluates blood pressure (BP) control at 24 months with self-management based on home BP monitoring plus self-titration of antihypertensive medication vs usual care among patients with poorly controlled hypertension in Spain.

## Introduction

Hypertension, or elevated blood pressure (BP), is the number one risk factor for ischemic heart disease and stroke, the 2 leading causes of death globally.^1,2^ Worldwide, management of high BP is suboptimal,^3^ including in Europe, where more than half of patients with hypertension are unable to achieve adequate BP control,^4^ despite the widespread availability of hypertension guidelines and the array of tools used to improve the delivery of long-term care, such as performance indicators, pay-for-performance schemes, and new information technologies.

The Chronic Care Model, an integrated framework widely adopted to guide the redesign of care based on the principles of patient-centeredness and evidence-based care, has proved to lead to better control of chronic conditions and to improve patient and economic outcomes.^5^ In the case of hypertension, evidence from randomized clinical trials and systematic reviews has shown that home BP monitoring (HBPM), when combined with other interventions (such as lifestyle changes, multidisciplinary care, telemonitoring, or medication self-titration) may reduce BP levels,^6,7,8,9,10,11,12,13^ although its effect appears to be strongly influenced by the intensity of cointerventions,^9,10,11,12,13,14,15,16,17^ with little to no effect on BP control when used alone.^9,17,18,19,20^ Overall, significant heterogeneity due to different inclusion criteria, organizational settings, self-monitoring regimens, the nature and intensity of cointerventions, target BPs in the included studies, or length of follow-up (with only 4 studies providing results at 12 months) calls for a cautious interpretation of the available evidence on hypertension self-management. Among these studies, 3 trials have addressed HBPM with medication self-titration, 1 from the US showing no effect at 6 months (in which the rate of medication self-titration was very low [approximately 20%]) and 2 from the UK showing a reduction in BP at 12 months.^13,14,15^ Moreover, routine clinical practice conditions may differ notably from randomized experiments with stricter follow-up routines; therefore, naturalistic evidence of the effect of HBPM and medication self-titration under routine clinical conditions and during extended periods is still missing.

We conducted the ADAMPA (Impact of Self-Monitoring of Blood Pressure and Self-Titration of Medication in the Control of Hypertension) randomized clinical trial to evaluate the effectiveness of an intervention including self-monitoring of BP plus self-titration of antihypertensive medication (based on an individualized prearranged plan) and educational components vs usual care (also with educational components) for reducing BP in patients with poor BP control. At 12 months, we did not find statistical differences in systolic BP (SBP; primary outcome measure) or in diastolic BP (DBP) between the intervention and control groups, although a higher percentage of participants in the intervention group achieved their recommended BP threshold compared with the control group.^21^ To gain additional knowledge on the effect of the intervention in the long term and under close to real-life conditions, we preplanned an additional 12-month extension of the study with passive follow-up. We present here the main findings of the ADAMPA trial at 24 months.

## Methods

### Study Design

The ADAMPA study is a pragmatic (ie, tries to mimic routine clinical practices as much as possible), randomized, unblinded clinical trial with 2 parallel groups assigned at a ratio of 1:1 to self-management (which includes educational components, HBPM, and self-titration of antihypertensive medication based on a patient’s family physician’s preestablished adjustment plan) or to usual care (also with educational components). The clinical research ethics committee of Hospital Clínico Universitario de Valencia approved the study protocol, as did the Spanish Agency for Medicines and Health Products. All participants provided written informed consent prior to enrollment in the study. None of the health professionals involved in the ADAMPA study received any payment for the recruitment and follow-up of patients or their participation in the study. The study protocol has been previously published^22^ and is available in Supplement 1. This study followed the Consolidated Standards of Reporting Trials (CONSORT) reporting guideline.

### Setting

The ADAMPA study was conducted in a Health District of the Valencia Health System (VHS), an extensive network of public hospitals and primary health care centers in the region of Valencia in Spain, covering about 97% of the region’s population (5 million inhabitants). The VHS is funded and provided by the Valencia regional government as a part of the Spanish national health system, a public, universal, and mostly free at the point-of-care, decentralized health care system.^23^ The VHS is geographically structured into 24 Health Districts, where each of the Health Districts includes a reference hospital and is subdivided into Primary Care Areas served by primary care centers.

### Participants

Patients aged 40 years or older, with a diagnosis of hypertension in their electronic medical record, with uncontrolled hypertension (with a mean SBP reading on the reference arm of >145 mm Hg or a DBP of >90 mm Hg on the baseline examination), and voluntarily agreeing to join the study were eligible for inclusion (see eAppendix 1 in Supplement 2 for details on exclusion criteria). Recruitment took place from July 21, 2017, to June 30, 2018; main outcomes were captured at 12 months, and an extension of the study was preplanned, collecting a reduced set of outcome variables with passive follow-up up to 24 months (study completion, August 25, 2020).

### Randomization and Allocation

**Figure 1.**
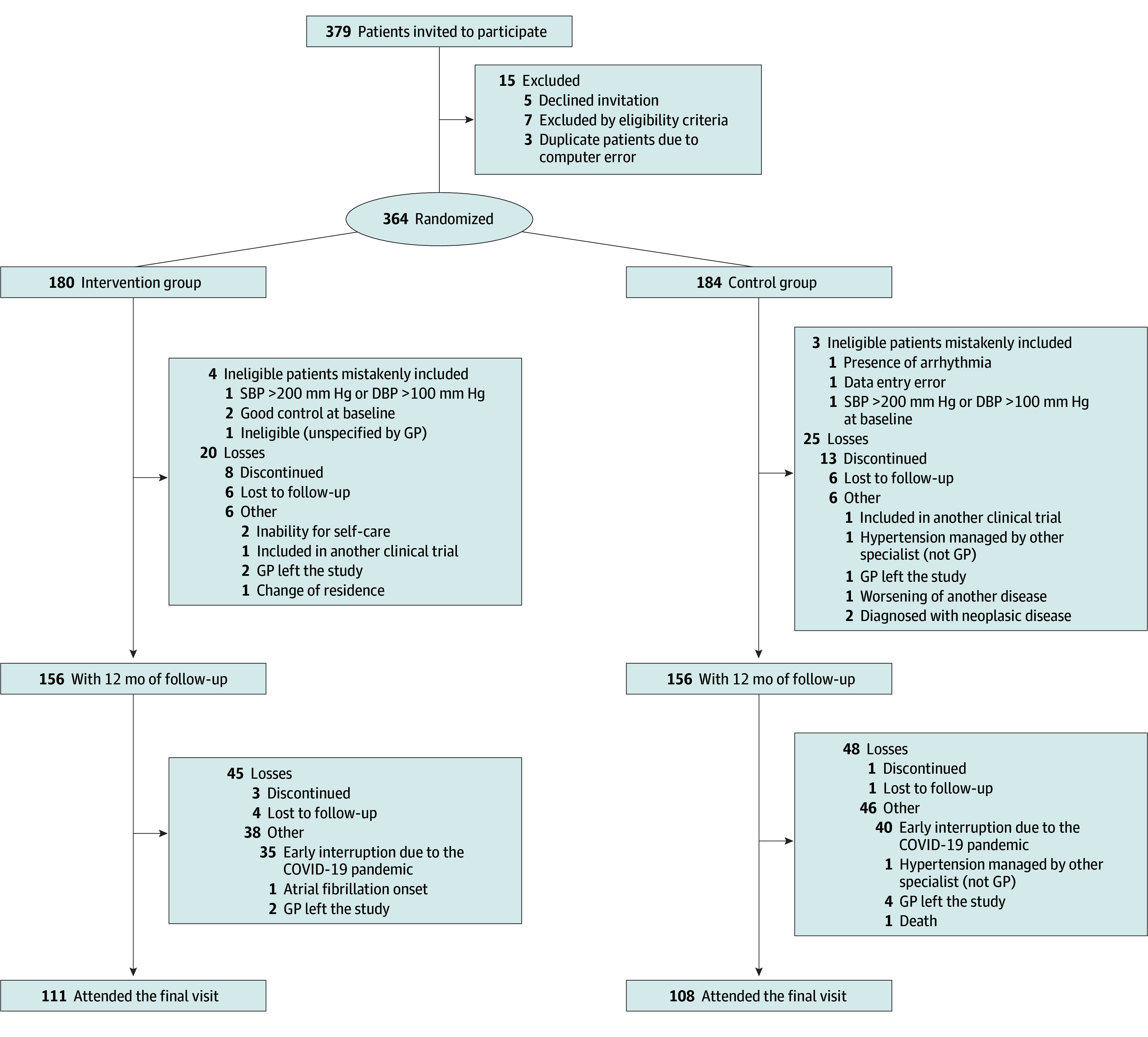
CONSORT Flow Diagram DBP indicates diastolic blood pressure; GP, general practitioner; and SBP, systolic blood pressure.

Potentially eligible patients were recruited by family physicians who performed a preliminary examination and obtained written informed consent. Patients were randomized at a 1:1 allocation ratio, with a centralized online randomization system assigning participants to usual care vs self-management. We used a minimization strategy^24^ considering age, sex, SBP higher than 160 mm Hg, and comorbidities (diabetes, cardiovascular disease, stroke, and chronic kidney disease). After randomization, a complete examination was scheduled by the research team (baseline visit); some patients either dropped out before that visit or were excluded because the examination revealed that they were not eligible and were mistakenly randomized. Losses to follow-up throughout the study and their reasons are detailed in Figure 1.

### Intervention and Control Groups

The intervention has been described thoroughly elsewhere.^21,22^ In short, family physicians defined individualized care plans and BP targets in conjunction with the patients, who received the recommendations for hypertension management from the clinical practice guidelines in force during the study period.^25,26^ Patients were provided with a booklet containing user-friendly, educational materials on how to improve BP control, written instructions for self-monitoring at home, sheets for recording BP readings every morning and afternoon during the first week (7 days) of every month, and instructions for self-adjustment of medication (dose adjustment or add-on medication) when needed based on BP readings using a color traffic light system (eAppendix 2 and eAppendix 3 in Supplement 2). Patients were trained to use and were provided with a validated home BP monitor (Omron M3 model HEM-7131-E; Omron Healthcare Inc). During the first 12 months of follow-up, patients were instructed to routinely register their BP readings, BP-related health encounters, and medication self-titration schemes in the allotted sheets of their booklets, and they were contacted by telephone to clarify possible doubts. Patients had to self-adjust their medication without any additional contact with their family physician, other health care workers, or coaches when their SBP or DBP was above the predefined target for 4 or more days in the measurement week, and they continued taking the modified therapeutic regimen for 3 weeks when an appointment with the physician was scheduled. At this visit, the therapeutic plan was updated. In the extension phase, in which nurses were involved, patients in the intervention group continued HBPM and medication self-titration. Nurses received information about the trial and were aware of which patients were in the intervention and control groups of the ADAMPA study, but they did not have any specific role apart from their usual practice. They treated patients (face to face or by telephone) in the context of routine care without preplanned follow-up visits and provided support to enhance patients’ empowerment based on patient-specific needs.

Participants in the control group received routine hypertension care (health education included) during the whole study. All relevant concomitant care within the routine clinical practice was at the discretion of the attending family physician.

### Outcomes

The main outcome in the extension phase was the adjusted mean difference (AMD) in SBP between the intervention and control groups at 24 months. Readings were taken by the family physician at baseline and at 6-, 12-, and 24-month follow-up visits, following measurement recommendations by the European Society of Hypertension and European Society of Cardiology (ESH–ESC) guidelines^25,26^ using a validated home BP monitor (Omron M3 model HEM-7131-E). However, the final 24-month visit had to occur early for some patients due to the relocation of their physicians to a different Health District before the end of the 24-month follow-up period, resulting in a median follow-up of 23.8 months (IQR, 19.8-24.5 months). Secondary outcomes assessed at the final visit were the AMD in DBP between groups, the difference in the percentage of patients achieving the BP target (SBP <140 mm Hg and DBP <90 mm Hg), health-related quality of life (measured using the EuroQoL-5D^27^), behavioral change (smoking, exercise, and body weight), use of health care resources, and occurrence of adverse events.

### Sample Size

A sample size of 382 patients was estimated to have 90% power to detect a mean (SD) difference of 5 (15) mm Hg in SBP between groups (primary outcome) with a 2-tailed contrast and an α error of .05, which represents a clinically relevant difference based on previous trials.^9,14,15^ For reasons unrelated to the study and detailed elsewhere,^21^ we were able to randomize 364 patients, of whom 312 completed follow-up at 12 months; 219 patients attended the final visit of the extension phase.

### Statistical Analysis

Statistical analysis was conducted from August 2022 to February 2024. First, we described the characteristics of the patients who remained in the study during the extension period, using the χ^2^ test for categorical variables and the *t* test for continuous variables. Second, in intention-to-treat analyses, we estimated the crude differences in SBP and DBP readings, with their corresponding 95% CIs, between baseline and 12 months and between baseline and the final visit, as well as the crude mean differences and the AMDs with their corresponding 95% CIs between groups in SBP and DBP at the final visit. Linear mixed-effects models were used to compare SBP and DBP between groups adjusting for sex, age, baseline SBP, obesity, and diabetes as fixed effects and for family physician as a random effect. Visual inspection of the residual plots did not show any major deviations from homoscedasticity or normality. Third, we performed several preplanned stratified analyses of between-group AMD in SBP at the final visit, with their corresponding 95% CIs, by sex, age group (40-64 years, 65-79 years, and ≥80 years), baseline SBP (<160 mm Hg vs ≥160 mm Hg), presence of diabetes and other comorbidities (cerebrovascular disease, peripheral artery disease, chronic kidney disease, angina, or acute myocardial infarction), diabetes plus elevated baseline SBP, obesity, overweight and obesity, and obesity plus elevated baseline SBP. Fourth, we estimated the proportion of patients achieving the BP target in the final visit using the recommendations established in the 2018 ESH-ESC guidelines,^26^ as well as the difference in proportions between groups. Fifth, we estimated differences between groups in the final visit for behavioral outcomes (smoking, obesity, and sedentary lifestyle), health-related quality of life, and use of health care resources. In addition, the occurrence of adverse events during the extension follow-up period was also assessed.

Sensitivity analyses were performed to assess the robustness of the primary end point analyses. First, multiple-imputation analysis was performed, using a model-based approach for missing data based on a Markov chain Monte Carlo simulation. Second, we reran the main analysis including patients with missing BP measurements at 24 months using the last observation carried forward for those whose last BP measurements were at 12 months. A 2-sided *P* < .05 was considered significant. Analyses were performed using Stata, version 14 (StataCorp LLC), and R, version 3.6.0 (R Project for Statistical Computing).

## Results

From the 312 patients attending the 12-month visit, 219 (70.2%; 111 in the intervention group and 108 in the control group) remained in the study during the extension period and provided BP readings at the final follow-up visit. Of the 93 patients who did not attend the final visit, 75 experienced an early interruption of the trial extension follow-up due to restrictions for attending health care centers during the COVID-19 pandemic, 4 abandoned the study, 5 were lost to follow-up, and 6 did not attend the final visit for other causes (Figure 1). Losses among study groups were similar.

Overall, 120 patients (54.8%) were women, 99 patients (45.2%) were men, the mean (SD) age was 64.3 (10.1) years, the mean (SD) baseline SBP was 155.6 (13.1) mm Hg, the mean (SD) DBP was 90.8 (7.7) mm Hg, 52 (23.7%) had diabetes, and 67 (30.6%) previously used HBPM (Table 1). No differences between groups were observed in the baseline characteristics of the patients reaching the final follow-up visit. Compared with the 93 patients who did not attend the final visit, the 219 patients who attended the 24-month visit had a higher mean (SD) baseline DBP (90.8 [7.7] mm Hg in the intervention group vs 88.6 [8.6] mm Hg in the control group; *P* = .03) and were more likely to be housewives at baseline (14.6% [32 of 219] in the intervention group vs 4.3% [4 of 93] in the control group), although most had permanent work or were retired in both groups (78.5% [172 of 219] in the intervention group vs 86.0% [80 of 93] in the control group) (eTable 1 in Supplement 2). Baseline characteristics by study group, for patients who attended the 24-month visit and for patients who did not, are shown in eTable 2 in Supplement 2.

**Table 1.** Baseline Characteristics of the ADAMPA Trial Patients Achieving the Final Follow-Up Visits

Characteristic	Patients, No. (%)		
	Total (N = 219)	Intervention (n = 111)	Control (n = 108)
Sex			
Female	120 (54.8)	61 (55.0)	59 (54.6)
Male	99 (45.2)	50 (45.0)	49 (45.4)
Age, mean (SD), y	64.3 (10.1)	65.1 (9.6)	63.4 (10.5)
Systolic blood pressure, mean (SD), mm Hg	155.6 (13.1)	155.3 (13.2)	156.0 (13.2)
Diastolic blood pressure, mean (SD), mm Hg	90.8 (7.7)	9.1 (7.9)	91.5 (7.4)
BMI			
Normal (18 to <25)	35 (16.0)	18 (16.2)	17 (15.7)
Overweight (25 to <30)	90 (41.1)	47 (42.3)	43 (39.8)
Obese (≥30)	93 (42.5)	45 (4.5)	48 (44.4)
BMI, mean (SD)	29.8 (4.9)	29.6 (4.9)	29.9 (4.9)
Educational level			
No qualification	12 (5.5)	7 (6.3)	5 (4.6)
Primary education	96 (43.8)	50 (45.1)	46 (42.6)
Secondary education	69 (31.5)	33 (29.7)	36 (33.3)
University degree or higher	42 (19.2)	21 (18.9)	21 (19.4)
Marital status			
Single	16 (7.3)	9 (8.1)	7 (6.5)
Married	152 (69.4)	75 (67.6)	77 (71.3)
Divorced	17 (7.8)	10 (9.0)	7 (6.5)
Widowed	34 (15.5)	17 (15.3)	17 (15.7)
Employment status			
Permanent work	67 (30.6)	31 (27.9)	36 (33.3)
Temporary work	1 (0.5)	0	1 (0.9)
Housewife	32 (14.6)	16 (14.4)	16 (14.8)
Unemployed	14 (6.4)	10 (9.0)	4 (3.7)
Retired	105 (48.0)	54 (48.7)	51 (47.2)
Smoking	47 (21.5)	20 (18.0)	27 (25.0)
Sedentary lifestyle	95 (43.4)	53 (47.8)	42 (38.9)
HRQoL (EQ5D score), mean (SD)	0.9 (0.2)	0.8 (0.3)	0.9 (0.2)
Comorbidities			
Diabetes	52 (23.7)	26 (23.4)	26 (24.1)
Cerebrovascular disease	7 (3.2)	3 (2.7)	4 (3.7)
Angina	2 (0.9)	1 (0.9)	1 (0.9)
Acute myocardial infarction	4 (1.8)	3 (2.7)	1 (0.9)
Peripheral artery disease	5 (2.3)	2 (1.8)	3 (2.8)
Chronic kidney disease	15 (6.9)	7 (6.3)	8 (7.4)
Years of onset hypertension, mean (SD)	10.8 (9.0)	11.0 (9.5)	10.6 (8.5)
No. of antihypertensive drugs, mean (SD)	1.7 (0.9)	1.8 (1.0)	1.6 (0.9)
No. of concomitant treatments, mean (SD)	2.9 (2.3)	2.7 (2.1)	3.2 (2.6)
Home blood pressure monitoring	67 (30.6)	31 (27.9)	36 (33.3)

Abbreviations: ADAMPA, Impact of Self-Monitoring of Blood Pressure and Self-Titration of Medication in the Control of Hypertension; BMI, body mass index (calculated as weight in kilograms divided by height in meters squared); EQ5D, EuroQoL-5D; HRQoL, health-related quality of life.

There was an increase in prescriptions of antihypertensive drugs in both groups at 24 months; the mean number of antihypertensive medications at 24 months was higher in the intervention group than in the control group (2.4 [95% CI, 2.2-2.5] vs 2.1 [95% CI, 1.9-2.3]; *P* = .04) (eTable 3 in Supplement 2). Of the 111 patients in the intervention group who reached the final follow-up visit, 76 (68.5%) made at least 1 medication adjustment, 43 (38.7%) increased the medication dose, and 61 (55.0%) added on a new drug at least once according to their self-titration plan, with a mean number of adjustments for these patients of 2.7 (95% CI, 2.2-3.3) (eTable 4 in Supplement 2). During the extension phase, 27.9% of patients in the intervention group (31 of 111) self-adjusted their medication.

At the final visit, BP decreased in both groups from baseline (SBP: intervention group, −21.3 mm Hg; 95% CI, −24.5 to –18.2 mm Hg; control group, −18.6 mm Hg; 95% CI, −21.8 to −15.5 mm Hg) and DBP (intervention group, −9.4 mm Hg; 95% CI, −11.2 to −7.7 mm Hg; control group, −8.6 mm Hg; 95% CI, −10.1 to −7.0 mm Hg). The AMD between groups at 24 months were −3.4 mm Hg (95% CI, –4.7 to −2.1 mm Hg; *P* < .001) for SBP and −2.5 mm Hg (95% CI, −3.5 to −1.6 mm Hg; *P* < .001) for DBP (Table 2 and eFigure in Supplement 2). The sensitivity analyses (using multiple imputation and carrying forward the last observation for those with missing BP measurements at 24 months) yielded similar results (eTable 5 and eTable 6 in Supplement 2).

**Table 2.** Systolic and Diastolic Blood Pressure at Baseline, 12 Months, and Final Follow-Up Visit of the Extension Phase

Group	Blood pressure, mean (95% CI), mm Hg^a^			Reduction, mean (95% CI), mm Hg			Difference between groups at 24 mo, mean (95% CI), mm Hg	*P* value
	At baseline	At 12 mo	At 24 mo	From baseline to 24 mo	From 12 to 24 mo			
**Crude systolic blood pressure**								
Intervention	155.3 (152.8 to 157.7)	136.0 (133.4 to 138.7)	133.9 (131.1 to 136.7)	−21.3 (−24.5 to –18.2)		−2.1 (−4.6 to 0.5)	−3.4 (−7.4 to 0.6)	.09
Control	156.0 (153.5 to 158.5)	139.2 (136.3 to 142.1)	137.3 (134.5 to 140.2)	−18.6 (−21.8 to −15.5)		−1.8 (−5.0 to 1.3)		
**Crude diastolic blood pressure**								
Intervention	90.1 (88.6 to 91.5)	81.3 (79.6 to 83.0)	80.6 (78.8 to 82.4)	−9.4 (−11.2 to −7.7)		−0.7 (−2.3 to 0.9)	−2.3 (−4.7 to 0.1)	.06
Control	91.5 (90.1 to 92.9)	83.4 (81.4 to 85.4)	82.9 (81.3 to 84.6)	−8.6 (−10.1 to −7.0)		−0.4 (−2.3 to 1.5)		
**Adjusted systolic blood pressure** ^b^								
Intervention	NA	NA	134.0 (133.0 to 134.9)^c^	NA		NA	−3.4 (−4.7 to −2.1)	<.001
Control	NA	NA	137.3 (136.4 to 138.3)^c^	NA		NA		
**Adjusted diastolic blood pressure** ^b^								
Intervention	NA	NA	80.2 (79.6 to 80.9)^c^	NA		NA	−2.5 (−3.5 to −1.6)	<.001
Control	NA	NA	82.8 (82.1 to 83.4)^c^	NA		NA		

Abbreviation: NA, not applicable.

^a^ All blood pressure measures correspond to the 219 patients attending the 24-month follow-up visit.

^b^ Adjusted for sex, age, baseline systolic blood pressure, obesity, diabetes (fixed effects), and general practitioner (random effect).

^c^ Mean estimate from the fitted model.

**Figure 2.**
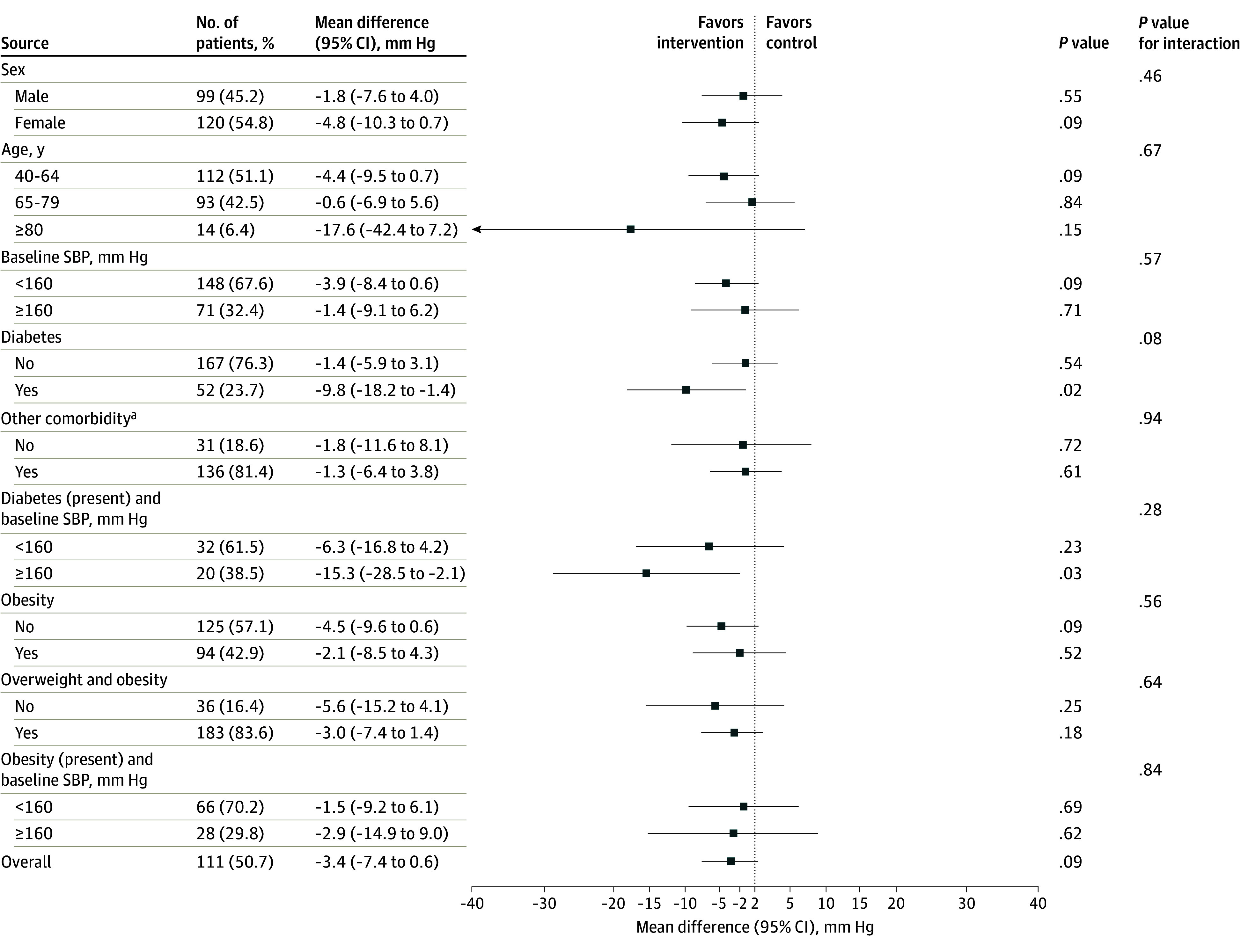
Forest Plot of the Stratified Analysis per Subgroups for the Extension Phase Among 219 Patients SBP indicates systolic blood pressure. ^a^Other comorbidities include cerebrovascular disease, peripheral artery disease, chronic artery disease, chronic kidney disease, angina, and acute myocardial infarction.

Stratified unadjusted analyses showed no differences in SBP among subgroups at 24 months. Greater differences were observed for patients with diabetes (−9.8 mm Hg [95% CI, −18.2 to −1.4 mm Hg]) and patients with diabetes and SBP at baseline of 160 mm Hg or higher (−15.3 mm Hg [95% CI, −28.5 to −2.1 mm Hg]), although interaction terms were not statistically significant (diabetes, *P* = .08 for interaction; diabetes and SBP ≥160 mm Hg at baseline, *P* = .28 for interaction) (Figure 2).

No differences were found with respect to the percentage of patients achieving the BP target at the final visit (64.0% [71 of 111] in the intervention group vs 53.7% [58 of 108] in the control group; difference, 10.3 [95% CI, −2.7 to 23.2]; *P* = .06) (eTable 7 in Supplement 2), the occurrence of adverse events, the change in behavioral risk factors, health-related quality of life, or use of health care resources (eTables 7-10 in Supplement 2), except for the mean (SD) number of visits to a primary care center without appointment, which was higher in the control group (0.2 [0.5] in the control group vs 0.06 [0.3] in the intervention group; *P* = .03) (eTable 10 in Supplement 2).

## Discussion

In this randomized clinical trial with a preplanned extension period of 12 months, with passive follow-up mimicking routine clinical conditions and a total follow-up close to 24 months, HBPM plus medication self-titration based on individualized care plans for patients with hypertension managed in primary care was associated with a reduction in SBP compared with usual care. An AMD of −3.4 mm Hg in SBP between groups at the final extension visit was observed, which, together with an AMD in DBP of −2.5 mm Hg, could result in clinical benefits if sustained over time; available evidence with up to 5 years of follow-up shows that similar BP reductions were associated with a 14% reduction in total cardiovascular events, a 28% reduction in strokes, and a 25% reduction in cardiovascular deaths.^28^ Our results at 24 months suggest a maintenance effect of the intervention to reduce BP (eFigure in Supplement 2), with similar results to those shown at 12 months of follow-up.^21^ Subgroup analyses for the primary outcome measure at the extension visit showed consistent results among subgroups. Although interaction terms were not significant, greater differences were observed among patients at higher risk, as seen in the TASMIN-SR (Targets and Self-Management for the Control of Blood Pressure in Stroke and at Risk Groups) study^15^ (as compared with the TASMINH 2 [Telemonitoring and Self-Management in Hypertension 2] study^14^); these differences could have clinically important implications, suggesting the potential of the intervention to reduce adverse outcomes among patients at higher risk. No other differences were found for the rest of the outcomes measured up to 24 months (percentage of patients achieving their BP targets, adverse events, health-related quality of life, behaviors, and use of health care resources), except for a slight reduction in the mean number of unplanned primary care visits in the intervention group.

Patient acceptance of the new treatment scheme, as well as an improvement in their capacity for self-management, may partly underlie the maintenance effect of the intervention for up to 24 months. Losses to follow-up in the extension phase were explained mainly by an early interruption of the trial extension follow-up due to restrictions for attending health care centers during the COVID-19 pandemic, rather than by lack of interest or patient commitment. Treatment intensity may be another relevant mediator. Overall, the mean number of antihypertensive medications increased in both groups from baseline, although it was slightly higher in the intervention group than in the control group at the end of the follow-up (2.4 vs 2.1). In addition to the treatment modifications made by their family physicians as part of routine care, 68.5% of patients in the intervention group attending the final visit self-adjusted their medication (eTable 4 in Supplement 2), a figure close to that of other studies.^14^ During the extension phase, 27.9% of patients in the intervention group self-adjusted their medication. Finally, professional engagement and other aspects that patient empowerment entails (such as more knowledge, higher skills, and confidence to actively participate in their own care) may have played an essential role in the achievement of consistent long-term results. We conducted qualitative research with family physicians and nurses involved in the study and found that, overall, both groups valued the intervention and were highly supportive of its implementation at a system level, reporting a shared perception of better BP control, the key role of patient empowerment, and reduced therapeutic inertia in the intervention group compared with usual care.^29^

Overall, HBPM as a single intervention has shown little or no effect in improving BP control. However, when combined with other interventions, such as interprofessional collaboration, education and training, active counseling, or medication titration strategies, relevant BP reductions have been observed, at least in the short term.^16,18,20,30,31,32^ To our knowledge, few randomized studies have addressed BP self-management,^10,33^ with only 1 trial to date assessing the effect of these interventions after 1 year.^10,34^ The ADAMPA trial showed results consistent with the aforementioned studies at 12 months^10,21,33^ and showed a maintenance effect beyond 1 year, consistent with the trial with long-term assessment.^34^ No increase in adverse events, decrease in quality of life, or intensification of health services use (beyond the increase in antihypertensive medication prescriptions) was observed at 2 years. Nonetheless, the ADAMPA trial at 24 months differs from the latter study^34^ in that it entailed a high degree of patient empowerment (mainly through self-adjustment of medication, without any kind of coaching) and telemonitoring was absent. The ADAMPA trial showed that a relatively simple and inexpensive intervention based on patient empowerment through HBPM and medication self-titration, with no telehealth components, was associated with a reduction in BP without an increase in adverse events or direct health care costs for up to 24 months.

## Limitations

Our study is subject to some limitations. First, 93 of the original 312 study participants (29.8%) attending the 12-month visit (main analysis) did not attend the 24-month visit; most of them (75 of 93 [80.6%]) experienced an early interruption of the trial extension follow-up due to restrictions for attending health care centers during the COVID-19 pandemic. Losses to follow-up were similar in both groups, and study participants attending the 24-month visit were also similar to the ones lost to follow-up. In addition, the sensitivity analysis including patients with missing BP measurements at 24 months using the last observation carried forward for those whose last BP measurements were at 12 months yielded similar results, suggesting a reduced potential bias. Second, the trial was unblinded to patients and professionals. This unblinding could lead to biases such as performance bias or the Hawthorne effect (where physicians and patients modify their behavior in response to being observed) or social desirability bias (patients overreporting positive behaviors or underreporting undesirable ones). Third, participating physicians managed patients in the intervention and control groups, which could result in a contamination bias, where some components of the intervention may have been extended to patients outside the intervention group of the study; if present, this effect would produce a result toward the null bias. Fourth, we did not evaluate the effect of HBPM on clinical outcomes. However, BP reduction has proved to be a reliable surrogate end point, highly correlated with reduced morbidity and cardiovascular mortality.^35,36,37^ Fifth, our study was designed for a single primary end point at 12 months; thus, all results from the 24-month extension presented here should be considered nonconfirmatory. Sixth, our results should be extrapolated with caution because we applied strict inclusion and exclusion criteria, resulting in some relevant subgroups omitted in our study (for instance, patients with adequately controlled hypertension, who account for approximately half of all patients with hypertension; patients with very high BP; or pregnant women).

## Conclusions

In this secondary analysis of a randomized clinical trial of self-management of BP including HBPM and self-titration of antihypertensive medication for patients with uncontrolled hypertension, a reduction in BP at 24 months was observed, with no increase in the use of health care resources or adverse events compared with usual care. These results suggest that simple, inexpensive, and easy-to-implement self-management interventions have the potential to improve the long-term control of hypertension in routine clinical practice.

## Notes

Supplement 1.
Study Protocol and Statistical Analysis Plan

Supplement 2. **eAppendix 1.** Exclusion Criteria **eAppendix 2.** HBPM and Self-Titration Instructions Sheets **eAppendix 3.** Individualized Adjustment Plan Sheet **eTable 1.** Baseline Characteristics of the ADAMPA Trial Patients Completing the 24-Month Visit and Patients Not Completing the 24-Month Visit **eTable 2.** Baseline Characteristics of the ADAMPA Trial Patients Completing the 24-Month Visit and Patients Not Completing the 24-Month Visit by Study Group **eTable 3.** Home Blood Pressure Monitoring and Medication Changes at 24 Months **eTable 4.** Self-Titration of Antihypertensive Medication in the Intervention Group (n=111) During the Whole Study Period and the Extension Phase **eTable 5.** Differences in Systolic and Diastolic Blood Pressure Among Study Groups at 24 Months, Crude And Adjusted: Sensitivity Analysis Including All Patients Attending the 12-Month Visit (n=312) Using Multiple Imputation **eTable 6.** Differences in Systolic and Diastolic Blood Pressure Among Study Groups at 24 Months, Crude and Adjusted: Sensitivity Analysis Including All Patients Attending the 12-Month Visit (n=312) Using the Last Observation Carried Forward **eTable 7.** Number and Percentage of Patients Achieving Their Target BP at the Final Follow-Up Visit in the Intervention and Control Groups **eTable 8.** Adverse Events During the Extension Follow-Up Period **eTable 9.** Behavioural Risks and Health-Related Quality of Life at 12 Months and at the Final Follow-Up Visit **eTable 10.** Health Care Services Utilization During the Extension Follow-Up Period **eFigure.** Mean SBP and DBP in Follow-Up Visits at Baseline, 6, 12 Months and the Final Follow-Up Visit for the Intervention (n=111) and Control Groups (n=108)

Supplement 3.
Nonauthor Collaborators

Supplement 4.
Data Sharing Statement
