# Supplementary material for: Long-Term Effect of Home Blood Pressure Self-Monitoring Plus Medication Self-Titration for Patients With Hypertension: A Secondary Analysis of the ADAMPA Randomized Clinical Trial
Source: JAMA Netw Open. 2024 May 10;7(5):e2410063. doi: 10.1001/jamanetworkopen.2024.10063 (PMC11087839; doi:10.1001/jamanetworkopen.2024.10063)
Supplement: Supplement 1. — Study Protocol and Statistical Analysis Plan [file jamanetwopen-e2410063-s001.pdf]

TITLE

Impact of Self-monitoring of Blood Pressure and Self-titration of Antihypertensive Medication in the Control of Hypertension and Adherence to Treatment. A Pragmatic, Randomized, Controlled Clinical Trial (ADAMPA Study).

1. Summary

Table **1. Summary of the trial characteristics**

Sponsor	INCLIVA
Title	Impact of Self-monitoring of Blood Pressure and Self-titration of Antihypertensive Medication in the Control of Hypertension and Adherence to Treatment. A Pragmatic, Randomized, Controlled Clinical Trial (ADAMPA Study).
Protocol Code	ADAMPA
Principal Investigators	José Sanfélix-Genovés<br>Centro de Salud de Nazaret<br>Departamento de Salud de Valencia Clínic-La Malvarrosa<br>Tel: 34 961925916<br>Email: sanfelix_jos@gva.es<br><br>Gabriel Sanfélix-Gimeno<br>Health Services Research Unit<br>Center for Public Health Research (CSISP-FISABIO)<br>Av. Catalunya 21. 46020 Valencia, Spain<br>Tel: 34 961925916<br>Email: sanfelix_gab@gva.es
Centers	Primary care centers from the Valencia Clínic-La Malvarrosa Health District, Consellería de Sanitat de la Comunitat Valenciana
IRB	The clinical research ethics committee from Hospital Clínico Universitario de Valencia (CEIC-HCUV)
Monitoring responsible	Spanish Clinical Research Network (SCReN).
Intervention	• Intervention group: The intervention consists of self-monitoring blood pressure at home, and subsequent medication self-titration, based on a medication adjustment plan pre-established by the family physician, in patients with uncontrolled hypertension.<br>• Control group: Patients in this arm will receive routine care for high blood pressure in the primary health care center.
Clinical trial phase	Phase IV
Objectives	To evaluate the comparative effectiveness of an intervention that includes educational components, self-monitoring of blood pressure and self-titration of antihypertensive medication in the improvement of control of hypertension compared to usual care in a poorly controlled population of hypertensive patients.
Design	Pragmatic, controlled, randomized, non-masked clinical trial with two parallel arms.
Disease	Hypertension
Primary outcome measure	Difference in mean systolic blood pressure, in mmHg. At 12 months of follow-up between the intervention and control groups, determined at physicians' practice with a validated automatic electronic sphygmomanometer.
Participants	Patients assigned to the Valencia Clinic-La Malvarrosa Health Department. Total number of patients to be randomized: 458 (229 per arm).
Treatment duration	12 months (also, a pragmatic extension with passive follow-up is planned for 24 months, collecting a reduced set of outcome variables, as secondary variables)
Calendar and expected completion date	The trial will take place over 3 years (6 months of recruitment, 12 months of follow-up for the main analysis of results, 12 months of pragmatic follow-up at 24 months, and finally 6 months for reporting results). Estimated Completion Date: 2020.

2. INDEX

1. SUMMARY
   Type of request
   Promoter Identification
   essay title
   protocol code
   Principal researchers
   Center(s) in which the trial is expected to be carried out
   Clinical Research Ethics Committee/s
   Name and qualification of the person responsible for monitoring
   Intervention and control groups
   Clinical trial phase
   Goals
   Design
   Study disease or disorder
   Primary endpoint
   Study population and total number of patients
   Treatment duration
   Schedule and expected completion date.
2. INDEX
3. GENERAL INFORMATION
   3.1. Identification of the trial
   3.2. Type of clinical trial
   3.3. Sponsor data
   3.4. Sponsor authorized person
   3.5. Investigators
      3.5.1. Principal investigators
      3.5.2. Co-investigators
      3.5.3. Clinical investigators
   3.6. Related institutions
4. BACKGROUND
5. HYPOTHESIS AND OBJECTIVES
   5.1. HYPOTHESIS
   5.2. OBJECTIVES
6. DESIGN
   6.1. Outcome measures
      6.1.1. Principal outcome measure
      6.1.2. Secondary outcome measures
   6.2. Design
   6.3. Measures to minimize or avoid bias
      6.3.1. Randomization
      6.3.2. Masking
   6.4. Intervention
      6.4.1. Intervention group
      6.4.2. Control group
   6.5. Patients follow-up
   6.6. Identification of the data to be recorded
   6.7. Study discontinuation criteria
   6.8. End of trial
7. SELECTION AND WITHDRAWAL OF PATIENTS
   7.1. Inclusion criteria

7.2. Exclusion criteria
7.3. Expected criteria for withdrawal of study subjects
7.3.1. Voluntarily drop out of the study
7.3.2. For non-compliance or violation of the rules contained in the protocol
7.3.3. Women who become pregnant during the follow-up period of the study
7.4. Procedures to Follow for Patients Leaving the Study
8. MEDICATION
8.1. Trial medication
8.2. Allowed medication
8.3. Acquisition, packaging and traceability of the medication used in the trial.
8.4. Schedule of visits and evaluations
8.5. Procedures for each visit
8.5.1. Selection visit
8.5.2. Baseline visit
8.5.3. Follow-up visit
8.5.4. Final visit
8.5.5. Treatment visit
9. EFFICACY ASSESSEMENT
9.1. Efficacy variables and evaluation methods
10. SAFETY ASSESSMENT
11. STATISTICAL ANALYSIS
11.1. Sample size calculation
11.2. Statistical analysis
12. ETHICAL ASPECTS
12.1. Ethics Committee (Helsinki) and Authorities
12.2. Informed consent
12.3. Data protection
12.4. Monitoring and auditing
12.5. Protocol Compliance
12.6. Early Termination or Suspension of the Study
13. FUNDING AND INSURANCE
13.1. Funding
13.2. Insurance
14. PUBLICATION POLICY
14.1. Publication of the study protocol and results
15. REFERENCES

ANNEX 1. Serious Adverse Event Reporting Form

3. GENERAL INFORMATION

3.1 Trial identification

Title: Impact of Self-monitoring of Blood Pressure and Self-titration of Antihypertensive Medication in the Control of Hypertension and Adherence to Treatment. A Pragmatic, Randomized, Controlled Clinical Trial (ADAMPA Study).

Protocol code: ADAMPA

EudraCT number: 2016-003986-25

Clinicaltrials.gov

3.2 Trial design

Pragmatic, controlled, randomized, non-masked clinical trial with two parallel arms.

3.3 Sponsor

Instituto de Investigación Sanitaria INCLIVA
Avd. Menéndez Pelayo, 4 Acc
46010 Valencia

3.4 Sponsor authorized person

Marta Peiró Signes
Scientific Subdirector INCLIVA

3.5 Investigators

3.5.1 Principal Investigators

José Sanfélix-Genovés
Centro de Salud de Nazaret
Departamento de Salud de Valencia Clínic-La Malvarrosa
Tel: 34 961925916
Email: sanfelix_jos@gva.es

Gabriel Sanfélix-Gimeno
Health Services Research Unit
Center for Public Health Research (CSISP-FISABIO)
Av. Catalunya 21. 46020 Valencia, Spain
Tel: 34 961925916
Email: sanfelix_gab@gva.es

3.5.2 Co-investigators

Salvador Peiró Moreno, Aníbal García Sempere, Isabel Hurtado Navarro, Manuel Ridao López, Clara Rodríguez Bernal (Health Services Research Unit, Center for Public Health Research (CSISP-FISABIO)).
Irene Marco Moreno, Patricia Martínez Ibáñez, Greta Borrás Moreno, Lucia Martínez Ibáñez, Eugenia Avelino Hidalgo, María Boveda García, Ignacio Barreira, Kate Herrero Mangley, Mercedes Calleja del Ser. Departamento Valencia Clínic- La Malvarrosa. Instituto de Investigación Sanitária INCLIVA.

3.5.3 Clinical researchers (MEFI)

Forty-seven researchers, clinical family doctors, distributed in 17 Basic Health Areas and in 21 Primary Care Centers (Health Centers and Auxiliary Centers). Department Valencia Clínic- La Malvarrosa. INCLIVA Health Research Institute.

3.6 Related institutions

Departamento de Salud Valencia Clínico Malvarrosa
Instituto de Investigación Sanitaria INCLIVA.
Fundación para el Fomento de la Investigación Sanitaria y Biomédica de la Comunidad Valenciana (FISABIO).

4. Background

The presence of hypertension is one of the most important issues in the global burden of disease (1). In developed countries, the degree of control of hypertension has increased progressively over the last 15 years and has contributed to a decline in cardiovascular morbidity and mortality (2-8). However, a recent study carried out in 12 European countries showed that more than 50% of patients treated for hypertension continued to have uncontrolled blood pressure (BP) (9) and that results are far from ideal. As a large part of hypertension management is carried out in primary care (PC) and it is one of the most prevalent problems encountered by General Practitioners (GP), interventions aimed at improving its management should preferably be made in this setting. Recent hypertension clinical guidelines put emphasis on self-measured blood pressure monitoring (SMBP) by patients and on team-based systems to manage the condition (10).

Self-measured blood pressure monitoring at home (SMBP) is practiced extensively nowadays. In the United Kingdom and Canada it is highly recommended by GPs and used by more than 30% of patients (11, 12). Systematic reviews have shown disparate information regarding the effectiveness of SMBP alone in reducing blood pressure (BP). On the other hand, self-monitoring in conjunction with co-interventions (including systematic medication titration by doctors, pharmacists, or patients; education; or lifestyle counseling) has been shown to lead to clinically significant BP reduction, which persists for at least 12 months. Nevertheless, the effectiveness of SMBP requires additional evaluation given that its definition in those studies is highly heterogeneous (different clinical protocols, different strategies for additional support and management) and given the fact that most studies have short follow-ups (1 year or less) (13,14).

Regarding home titration of antihypertensive medication, evidence is more limited and shows mixed results. Two clinical essays, the TASMINH2 (15) and the TASMINH-SR (16), both in the United Kingdom and developed in the primary care setting by the same research team, are some of the most recent and interesting clinical trials carried out in this field. In these studies, SMBP together with self-titrate medications (according to a previously agreed plan), combined with telemedicine components, was compared with usual care. In both studies systolic blood pressure (SBP) decreased from baseline to 12 months, with significant differences between the intervention and control group (5.4 and 9.2 mmHg, respectively). Frequency of side effects was similar in both groups (15,16). The TASMINH-SR study is of special relevance because it was carried out with high-risk patients (with a personal history of stroke, ischemic cardiopathy, diabetes or kidney failure), a population of special interest to achieve BP targets (16). On the other hand, a clinical trial carried out in the US in a low-income, predominantly minority population, aimed to determine whether health coaching, SMBP and home titration of antihypertensive medications could improve BP control compared with SMBP and health coaching alone. The results showed that both the home-titration arm and the no–home-

titration arm had a reduction in SBP, with no significant differences between them from baseline to 6 months (17).

Finally, when interpreting hypertension studies over time, it is important to proceed with caution, as the definition of the condition changes almost with every update of guidance. For instance, earlier versions of guidelines such as those of the Joint National Committee (JNC) and of the European Society of Hypertension (ESH)/European Society of Cardiology (ESC), suggested more restrictive BP control objectives than recent versions (especially in patients over 60 years old, diabetics and patients with renal failure)(18-20). These objectives may be modified again in the light of the results of recent studies (21-23).

5. HYPOTHESIS AND OBJECTIVES

5.1 Hypothesis

The starting hypothesis of the trial is that an intervention of self-monitoring and self-adjustment of the medication in hypertensive patients may allow an improvement in the management of these patients with respect to usual clinical practice.

5.2 Objectives

1. To evaluate the effectiveness of an intervention based on self-monitoring and self-adjustment of medication in poorly controlled hypertensive patients.

2. Evaluate its impact on adherence to treatments, lifestyles, quality of life, the occurrence of adverse events, the use of health services, and the costs of care.

3. Estimate its efficiency (cost-effectiveness).

4. Evaluate the views and experiences of patients, caregivers and health professionals about self-management, self-monitoring and self-adjustment of antihypertensive medication

6. DESIGN

6.1 Outcome measures

6.1.1 Primary outcome meassure

Difference in mean systolic blood pressure, in mmHg. At 12 months of follow-up between the intervention and control groups, determined at physicians' practice with a validated automatic electronic sphygmomanometer.

At least 2 measurements should be taken in the sitting position, spaced 1-2 minutes apart, or an additional measurement if the first 2 are quite different. The average of the 2 measurements taken as valid will be considered as TA[8].

6.1.2 Secondary outcome meassures

1. Difference in mean systolic blood pressure, in mmHg at 6 and 24 months of follow-up between intervention and control groups determined at physicians' practice with a validated automatic electronic sphygmomanometer.

2. Difference in mean diastolic blood pressure, in mmHg at 6, 12 and 24 months of follow-up between intervention and control groups determined at physicians' practice with a validated automatic electronic sphygmomanometer.

3. Percentage of patients with SBP <140 mmHg and DBP<90 mmHg at 6, 12 and 24 months of follow-up.

4. Quality of life (as measured by EuroQoL-5D) at 6, 12 and 24 months of follow-up.

5. Adherence measured by proportion of days covered (PDC) at 6 and 12 months of follow-up.

6. Persistence, defined as period of continuous use of the corresponding drug from the beginning of the follow-up until its discontinuation at 6 and 12 months of follow-up.

7. Therapeutic inertia (TI), defined as the number of patients whose pharmacological treatment had not been modified, divided by the number of patients not reaching the target values (SBP and/or DBP measurements taken at 6 and 12 months of follow-up), according to the recommendations of the European Society of Hypertension and European Society of Cardiology (19).

Other outcome meassures:

1. Changes in lifestyle (smoking, exercise, body weight) at 6, 12, and 24 months compared to these characteristics at baseline.

2. Clinical events: We will assess if any of the following adverse events are present during the follow-up: angina, myocardial infarction, stroke, hypotensive crisis and death.

3. Use of health services for hypertension at 6, 12 and 24 months.

4. Incremental cost per quality-adjusted life year gained in the intervention group compared to the control group.

5. Views and experiences of patients and health professionals on the self-management (SMBP plus self-titration) of hypertension.

6.2 Design

Pragmatic, controlled, randomized, non-masked clinical trial with two parallel arms.

6.3 Measures to minimize or avoid bias

6.3.1 Randomization

Once the patients have received the study information and signed the corresponding informed consent, they will be randomized to usual care or to the intervention-self-monitoring and self-titration group (1:1 ratio) using a computerized randomization system. A randomization process with minimization will be carried out to preserve a balance between groups in variables considered key a priori by the research team. The minimization criteria will be: age, gender, SBP >160 mm HG, diabetes, cardiovascular disease (ischemic heart disease, heart failure, cardiomyopathy, and peripheral arterial disease), cerebrovascular accident (stroke), and chronic kidney disease.

6.3.2 Masking

Due to the nature of the intervention, it is not feasible to blind the study for either the patients or the Research Family Physicians (MEFI), who are responsible for the patients. Likewise, the Collaborating Research Physicians (MIC), who will conduct the training interventions in the first year and collaborate with MEFI in follow-up visits, cannot be blinded

6.4 Intervention

6.4.1 Intervention group

The trial intervention is not pharmacological; instead, it involves modifying usual clinical practice. The intervention for self-monitoring and self-adjustment of medication consists of three key elements:

1) Patient training: This involves providing information and training on arterial hypertension, its associated risks, its management, the benefits of achieving adequate control, and general measures for improved control.
2) Training for self-monitoring: Patients will receive instruction on the correct method for ambulatory blood pressure monitoring (AMPA), as well as guidance on how to measure their blood pressure and record the values using the provided tools.
3) Training for self-adjustment: Patients will be educated about target blood pressure goals, individually tailored to each patient. They will also learn how to respond when faced with specific blood pressure readings, including the self-adjustment of their pharmacological treatment.

6.4.2 Control group

Patients assigned to the control group will receive information about hypertension and the importance of its appropriate management (as outlined in point 1 of the intervention). The control group will not undergo any additional interventions to simulate routine clinical practice management.

6.5 Patients follow-up

The follow-up of the patients will be conducted by the MEFIs in line with routine clinical practice conditions. MEFIs will be responsible for patient selection and inclusion in the trial and will execute the training actions as outlined in point 6.4.

Additionally, the MIC team will reinforce the training and information actions specified by the MEFI and defined in point 6.4. However, it's important to note that the MIC team will not make clinical decisions or modify the management or recommendations of the MEFI. They will also handle data collection during the scheduled visits in the study, including the baseline visit and follow-up visits at 6 and 12 months, working in collaboration with the MEFI.

In the first 12 months of the study, the MIC team will conduct bi-monthly telephone reinforcement sessions. These sessions aim to contact patients in the intervention group with the goal of improving adherence to the intervention and addressing any potential concerns.

In the second year (passive or pragmatic follow-up), patient follow-up will be aligned as closely as possible with routine management in usual clinical practice. Therefore, the role of the MIC will be carried out by a nurse

6.6 Identification of the data to be recorded

The MEFIs, in collaboration with the MICs, will be responsible for collecting data at each of the Health Centers during the baseline and follow-up visits. The MICs will work in agreement and in cooperation with the MEFIs. It's important to note that the MICs will not make any clinical decisions regarding the patients participating in the study; this responsibility solely rests with the MEFI.

In cases where, during the baseline or follow-up visits, the MICs obtain additional health-related information from the patients (such as medical history or comorbidities) or acquire more recent study variables than those recorded in the patient's electronic medical record (e.g., BMI, blood pressure), they will include this information in the patient's clinical history. To facilitate this process, a meta-profile will be created (ADAMPA study), ensuring that all patient information (study variables) used in the trial is accurately reflected in the patient's medical record.

The data will be recorded in the visits -baseline, at 6 months, the final at 12 months and the extension at 24 months in the Data Collection Notebook (CDR) prepared for this purpose. The CDR will be completed in computer support. The sources to obtain the necessary data for the study will be:

- The electronic medical record of the Ambulatory Information System –SIA- (ABUCASIS) available in all Health Centers.
- The ORION program that allows access to the electronic hospital medical record.
- The clinical interview with the patient at the established visits (baseline, at 6, 12 and 24 months).
- Information on paper: serious adverse events and self-monitoring and self-adjustment booklet.
- Additional information will be extracted from the Valencia Health System electronic databases: Minimum Basic Data Set (MBDS), ABUCASIS and the Population Information System (SIP).

The following information will be collected at each visit.

Table 2. Data collection throughout the trial

**Visita basal**	**Visitas 6 y 12 meses**	**Visita 24 meses**
- BP*<br>- Sociodemographic information<br>- Lifestyle habits**<br>- Comorbidities<br>- Duration of hypertension<br>- Antihypertensive treatments<br>- number of concomitant treatments<br>- Health services use***<br>- Quality of life (EQ5D)	- BP*<br>- Lifestyle habits**<br>- Health services use***<br>- Adverse events<br>- Antihypertensive treatments<br>- number of concomitant treatments<br>- Quality of life (EQ5D)	- BP*<br>- Lifestyle habits**<br>- Health services use***<br>- Adverse events<br>- Antihypertensive treatments<br>- Quality of life (EQ5D)

*Blood pressure; ** Smoking habit, exercise, BMI; ***Information relative to the previous year. Its determination will be made at the health center with a validated automatic electronic sphygmomanometer. At least 2 measurements will be taken in a seated position, spaced at 1-2 minutes, and another additional measurement if the first 2 are very different. The average of the measurements taken will be recorded (8).

6.7 Study discontinuation criteria

Based on the study's characteristics and considering previous experiences, there is no anticipation of needing to interrupt the study due to safety concerns or lack of efficacy. Consequently, there will be no interim analysis conducted for these purposes, nor is the presence of a dedicated safety committee required. If any serious adverse events do arise, the trial research team will be responsible for their evaluation.

6.8 End of trial

The trial will conclude on the date of the last scheduled visit, with an anticipated total follow-up period of 24 months. Primary results analysis is planned at the 12-month mark. The subsequent 12 to 24 months will serve as a pragmatic extension with passive follow-up, excluding telephone reminders by the collaborating researchers. A final visit for patients remaining in the study will capture a reduced set of outcome variables (secondary endpoints).

7. SELECTION AND WITHDRAWAL OF PATIENTS

7.1 Inclusion criteria

- 40 years or older
- Diagnosis of hypertension of any origin
- Systolic blood pressure (SBP) > 145 or diastolic blood pressure (DBP) > 90 mm Hg at baseline
- Voluntarily participation in the study and having signed the corresponding informed consent.

7.2 Exclusion criteria

- Inability to understand and/or perform self-adjustment of the medication, including dementia or significant cognitive impairment (at the discretion of the investigator performing the recruitment).
- History of orthostatic hypotension (fall> 20 mm Hg in SBP after adopting the orthostatic position).
- SBP> 200 or DBP> 100 mm Hg on the baseline examination
- Being on treatment with more than 4 antihypertensive drugs
- Participation in another study on high blood pressure or in a clinical trial
- Presence of tremor or neurological disease that makes it difficult to perform BP self-measurement.
- Presence of arrhythmia
- Presence of terminal illness
- Chronic disability impeding to leave home
- History of acute cardiovascular event in the last 3 months
- Hypertension managed directly by specialist physicians outside the primary care setting.
- Spouse selected for the study

- Non-resident or transient patients
- Pregnancy

7.3 Expected criteria for withdrawal of study subjects

7.3.1 Voluntarily drop out of the study

7.3.2 For non-compliance or violation of the rules contained in the protocol

Participating patients will have the right to withdraw from the study at any time. Additionally, the investigator may discontinue a patient from the study if she deems it necessary for any reason, including:

- Ineligibility (retrospective if not detected at enrollment)
- an adverse event leading to inability to fulfill with trial procedures
- disease progression leading to inability to fulfill with trial procedures
- withdrawal of consent
- lost to follow-up

7.3.3 Women who become pregnant during the follow-up period of the study

Since the study includes individuals over the age of 40, there is a possibility of pregnancy during the follow-up period. However, due to the naturalistic conditions of the study, it is not deemed necessary to mandate contraceptive measures for women of childbearing age. Doing so could introduce additional risks. It's important to note that the ADAMPA protocol does not involve drug administration, and any risks associated with drug treatments are unrelated to the study. These risks would arise from medications prescribed by a participant's doctor, regardless of their involvement in the study. Nonetheless, pregnancy can complicate the management of hypertension and vice versa, necessitating specific and distinct medical monitoring beyond what's outlined in the intervention protocol. Consequently, if a participant becomes pregnant during the follow-up period, they will be excluded from the assigned protocol. Instead, their care will be overseen by their doctor(s), while still maintaining their endpoint follow-up and inclusion in the intention-to-treat analysis.

7.4 Procedures to Follow for Patients Leaving the Study

We will employ an intention-to-treat approach for outcome analysis. This means that all patients will be included in the analysis based on their randomized arm, even if they discontinue the trial, do not fully adhere to the intervention, or deviate from the protocol. All participants, including those who face these challenges, will be invited to attend follow-up visits at 6, 12, and 24 months. In cases of complete loss of a patient, they cannot be included in the primary analysis since the main outcome measure will not be available. If a participant abandons the study, we will document the reason in the Case Data Report (CDR) whenever possible.

8. MEDICATION

8.1 Trial medication

The ADAMPA study is designed to assess the effectiveness of an intervention that modifies usual clinical practice (self-monitoring and self-adjustment intervention versus usual care). It's important to note that the study does not intend to evaluate the effectiveness or safety of

medications. All medications, including antihypertensives and other therapeutic groups, will be prescribed based on the criteria of the family doctor in routine clinical practice conditions,.

8.2 Allowed medication

All medications, both antihypertensives and other therapeutic groups, will be prescribed according to the family doctor's criteria in routine clinical practice conditions.

8.3 Acquisition, packaging and traceability of the medication used in the trial.

As outlined in section 8.1, all medications, including antihypertensives and other therapeutic groups, will be prescribed in accordance with the criteria of the patient's family doctor, following routine clinical practice. No special conditioning or traceability will be conducted.

Patients will obtain their medications directly from pharmacies with the appropriate official medical prescription.

Nevertheless, the trial drugs' traceability is ensured through electronic prescription and dispensing systems. This technology will allow us to track both the prescriptions issued and the dispensing of medications at pharmacies throughout the trial's duration.

8.4 Schedule of visits and evaluations

The baseline visit will be made to the patients after selection by their responsible physician. Additionally, patients will be visited at 6 and 12 months. A last visit will be made at 24 months to the patients who continue in the study. The data that will be collected at each time point is shown in **Table 2** of section 6.6.

8.5 Procedures for each visit

8.5.1 Selection visit

Potential patient candidates for participation in the study will be selected opportunistically during medical consultations (case-finding) by the physicians responsible for the patients, known as Family Physician Investigators (MEFI). Ideally, MEFIs will aim to select one patient per day, every day of the week, until they reach the required number of patients for each MEFI.

During the same medical consultation at the Healthcare Center, MEFIs will inform patients about the study's objectives. If patients meet the inclusion criteria and none of the exclusion criteria (confirmed through electronic medical records and discussions with the patient and at the discretion of the doctor), they will be provided with an information sheet and an informed consent form. Patients will be given a private room to carefully review these documents at their own pace, with ample time provided if needed, and any questions they have will be answered.

To confirm the inclusion criteria related to hypertension (systolic blood pressure >140 mm Hg or diastolic blood pressure >90 mm Hg), the MEFI will measure blood pressure using a validated sphygmomanometer in accordance with the recommendations of the Guidelines for the management of arterial hypertension provided by the Task Force for the management of arterial hypertension of the European Society of Hypertension and the European Society of Cardiology[8] (**Table 3**).

Table 3. MEASUREMENT OF BLOOD PRESSURE IN THE OFFICE

When blood pressure (BP) is taken in the office, the following considerations should be taken into account
The patient must remain seated for 3-5 minutes before taking the blood pressure.
At least 2 measurements should be taken in the sitting position, spaced 1-2 minutes apart, or an additional measurement if the first 2 are quite different. The average of the 2 measurements taken as valid will be considered as TA.
Use standard sleeves but also have larger and smaller sleeves for wider and smaller arms.
Place the cuff at the level of the heart, whatever the position of the patient.
Take the BP in both arms at the first visit to detect possible differences. If they are, take the arm with the highest BP as the reference arm.
Measure the first BP after standing for 1 to 3 minutes in elderly, diabetic or other patients with suspected orthostatic hypotension.

Modified from Mancia G et al. J Hypertension. 2013.

For each patient, once it has been verified that the inclusion criteria are met and the exclusion criteria are not met, and once the informed consent is signed, the MEFI will enter into the web system and under mandatory guarantees of information security, the information of identification of the patients, marking the boxes corresponding to meeting the inclusion criteria and not meeting the exclusion criteria, and the information necessary for randomization with minimization (see 6.3.1 for more details on the minimization process). The MEFI will receive the result of the randomization and the anonymization code assigned to the patient via the Web, and the patient will be included in an electronic patient identification form with restricted access to Collaborating Research Physicians (MIC). The patient identification form will not contain any health data or other sensitive information. All the documents in which the data of the patients in the framework of the ADAMPA study are recorded (CDR, Patient's Notebook) will be identified only by the code assigned to each patient. Likewise, and in order to help their adequate follow-up and identification in the consultation, it will be indicated in the electronic medical record that the patient participates in the ADAMPA STUDY.

All patients, both those randomized to the control group and those in the intervention group, will receive information from the MEFI that a collaborating research physician (MIC team) will contact them by telephone to schedule the baseline visit.

Furthermore, the MEFI will provide all patients with an ADAMPA study Patient's Notebook. On the first page, the assigned patient code will be recorded. In the case of the intervention group, the MEFI will also note on the first page the arm in which the patient's blood pressure will be measured and their target figures. The Intervention Group Notebook will contain:

- First sheet: patient anonymization code, arm for blood pressure measurement, target figures
- Information about the importance of hypertension and lifestyle advice
- Instructions on how to take your blood pressure at home
- Instructions on how to act based on the voltage figures at home
- The medication self-adjustment plan established by the MEFI. The MEFI may establish the initial self-adjustment plan on that same visit or on another scheduled visit agreed upon with the patient.
- Sheets for recording BP values and for recording care contacts with your Health Center.

In turn, the Patient's Notebook for the control group will contain:

- First sheet: patient anonymization code, arm for taking blood pressure
- Information about the importance of hypertension and lifestyles
- Sheets for recording care contacts with your Health Center

8.5.2 Baseline visit

- Intervention group: the baseline visit will include the training intervention (described in section 6.4) and data collection (contained in Table 2 of section 6.6). Additionally, they will be informed that periodic group sessions will be held with the patients (in charge of the MICs) to reinforce the BP measurement technique, its recording and the self-adjustment plan.
- Control group will consist solely of data collection (consisting in **Table 2** of section 6.6). They will be told that they must follow their usual medical and nursing check-ups.

All patients, regardless of group, will be provided with a follow-up date at 6 months, informing them that they will be reminded beforehand by telephone and that they must come with the completed Patient Notebook.

If the patient does not attend the baseline visit, the patient will be located by telephone to give a new visit date or, in the event that they are not located or abandon the study, the date and the reason for it will be recorded.

The estimated period for the recruitment and first visit of the patients in the sample is estimated at 3 months.

Any data collected from patients that does not appear in their medical history will be added.

8.5.3 Follow-up visits

- 6-Month Visit:
    - Researchers will record data related to the variables of the follow-up visit (as outlined in **Table 2** of section 6.6). Data sources will include information provided by the patient, the Patient's Notebook, and the patient's electronic medical record.
    - All patients, regardless of their group, will receive a 6-month follow-up appointment and will be informed that they will receive a reminder call beforehand. They should also bring their completed Patient's Notebook.
- 12-Month Visit:
    - Researchers will collect data related to the variables of the follow-up visit (as detailed in **Table 2** of section 6.6), using information from the patient, the Patient's Notebook, and the electronic medical record.
    - Patients, irrespective of their group, will be informed that they will continue with their group-specific trial instructions (self-monitoring/self-adjustment or usual care). Simultaneously, they will maintain their regular medical and nursing appointments. They will also be informed that they will receive a phone call for a final visit within a year and should bring their completed Patient's Notebook.
- 24-Month Visit:
    - During this visit, a reduced set of outcome variables will be collected, including blood pressure, quality of life, and use of healthcare services (as outlined in **Table 2** of section 6.6).
    - The purpose of this visit is to conduct secondary analyses after a period of passive follow-up. This aims to evaluate adherence to the intervention under conditions even closer to usual clinical practice. Additionally, results at 24 months will be obtained during this visit.

- During the period from months 12 to 24, MICs will not provide reminders, and nurses will carry out complementary assistance work alongside MEFIs for follow-up, adjustment plan renewals, etc.

If the patient does not attend the follow-up visits, the patient will be located by telephone to give a new date of visit or, in the event that he is not located or leaves the study, the date and the reason for it will be recorded. Any data collected from patients that does not appear in their clinical history will be included in it.

<u>8.5.4 Final visit</u>

The visit at 12 months is the visit considered the final visit in the strict sense of the trial and with which data the main analyzes will be carried out. The 24-month visit will be carried out after passive follow-up to assess adherence (and effectiveness) to the intervention in conditions even closer to usual clinical practice.

<u>8.5.5 Treatment visit</u>

Visits to modify treatment, if applicable, will be carried out in the usual context of clinical practice by the patient's family doctor (MEFI) in both the control and intervention groups.

Additionally, the patients of the Intervention group will make visits or telephone consultations to their MEFI when so indicated according to the mode of action according to blood pressure figures, in the event that they must proceed to self-adjust the medication according to the plan. self-adjustment established by the MEFI and registered in the patient's notebook.

In the event that a patient from the intervention group, and based on their objective figures and the instructions received, has to proceed to the self-adjustment at home, they will again follow the mandatory instructions and that are also included in the Patient's Notebook and You will request an appointment with your MEFI within a maximum period of three weeks. On said visit after self-adjustment, the MEFI will review the patient's condition and renew the self-adjustment plan, recording said new self-adjustment plan in the Patient's Notebook.

9. EFICACY ASSESSMENT

<u>9.1 Efficacy variables and evaluation methods</u>

Table 4. Efficacy variables (comparative effectiveness) of the ADAMPA study

**VARIABLES**	**EVALUATION METHODS**
**Primary oucome measure**	
Difference in mean systolic blood pressure (SBP), in mmHg at 12 months of follow-up between the intervention and control groups.	It will be determined at physicians' practice with a validated automatic electronic sphygmomanometer.<br>At least 2 measurements will be taken in a seated position, spaced at 1-2 minutes, and another additional measurement if the first 2 are very different. The average of the measurements taken[8] will be considered as SBP/DBP.
**Secondary oucome measures**	
Difference in mean SBP, in mmHg at 6 and 24 months of follow-up between the intervention and control groups.	It will be determined at physicians' practice with a validated automatic electronic sphygmomanometer.<br>At least 2 measurements will be taken in a seated position, spaced at 1-2 minutes, and another additional measurement if the first 2 are very different. The average of the measurements taken[8] will be considered
Difference in mean diastolic blood pressure (DBP), in mmHg at 6, 12 and 24 months of follow-up between the intervention and control groups.<br>It will proceed in the same way and at the same time	

as with the SBP	as SBP/DBP.
Difference in the percentage of patients with SBP <140 mmHg and DBP <90 mmHg at 6, 12 and 24 months of follow-up between the intervention and control groups.	
Health-related quality of life (HRQoL) at 6, 12 and 24 months of follow-up.	The EuroQoL-5D questionnaire will be used to assess the impact of the intervention on the quality of life of the patients compared to the control group.
Between-group difference in Proportion of days covered with medication (PDC)	Secondary adherence (PDC and persistence) will be estimated using the prescription and dispensing data obtained from the Department's databases and the definitions detailed in section 6.1 will be applied.
Difference Between Groups in Persistence	
Difference between groups with respect to Therapeutic Inertia (IT)	Quotient between the number of patients whose pharmacological treatment has not been changed divided by the number of patients with SBP and/or SBP measurements taken in consultation at 6, 12, and 24 months of follow-up above the values target according to the recommendations of the European Society of Hypertension and European Society of Cardiology[8].
**Other outcome measures (tertiary endpoints)**:	
Differences in changes in lifestyles (smoking, exercise, weight) at 6, 12, and 24 months with respect to the baseline measurement.	Changes in lifestyles will be evaluated based on the information collected at follow-up visits.
Difference between groups in relation to the occurrence of adverse events: angina, myocardial infarction, stroke, hypotensive crisis and death.	Hospitalizations due to adverse events will be collected at follow-up visits. Outpatient and emergency visits due to hypotensive crises will also be collected.
Differences in the use of health services during follow-up (6, 12 and 24 months).	Including HTA-related primary medical and nursing care consultations (at the center by appointment or without an appointment, by telephone and at home). Visits to emergency hospital services, hospital admissions, hospitalization days per admission and main diagnosis
Incremental cost per additional quality-adjusted life year gained in the intervention group compared to the control group.	Cost-consequence analysis and cost-effectiveness analysis
Assessment of the intervention by the patients and the research family physicians.	Focus groups with patients and professionals involved (MEFI, MIC and nurses)

Complementary tests (functional, laboratory, radiological, etc.) in addition to those carried out in regular clinical practice to assess efficacy will not be performed.

10. SAFETY ASSESSMENT

The ADAMPA study is a clinical trial carried out under naturalistic conditions where the intervention is based on self-monitoring and self-adjustment of medication in poorly controlled hypertensive patients. The antihypertensive drugs used will be those that patients are taking as part of their usual medication. It is considered a low-risk clinical trial, and no differences in adverse events are expected compared to usual medical practice. Therefore, the need for a safety committee has not been anticipated. The reference safety information will be the technical data sheets of each of the antihypertensive drugs. Consequently, it is the investigator's responsibility to detect and document any event that meets the criteria and definitions of adverse events (AE) or serious adverse events (SAE), especially when such events are not described in the technical sheet.

During the study, the existence of adverse events, whether serious or not, will be checked according to the definitions provided in this section of the protocol. The Sponsor will be responsible for promptly notifying Health Authorities, such as the Spanish Agency for Medicines and Health Products (AEMPS), and Autonomous Communities (CCAA) if applicable, about serious and unexpected adverse reactions (RAGI), annual reports, or any other relevant

safety information. Additionally, the Sponsor will be responsible for notifying researchers about safety information.

a) Definitions:
Adverse event (AE Refers to any harmful incident to a patient or clinical trial subject treated with a drug, even if it doesn't necessarily have a causal relationship with the treatment.

Adverse reaction (AR): Denotes any harmful and unintentional reaction to an investigational drug, regardless of the administered dose.

Serious adverse event (SAE): Encompasses adverse events that, at any dose, cause death, threaten the subject's life, require hospitalization or its prolongation, result in permanent or significant disability, disability, or give rise to a congenital anomaly or malformation. For notification purposes, suspected adverse events considered important from a medical perspective will also be treated as serious, even if they don't fully meet the above criteria.

Serious and Unexpected Adverse Reaction (GUAR): Represents an adverse reaction whose nature or severity doesn't correspond to the information in the product's technical data sheet.

Causality criteria:
The establishment of a causal relationship between the antihypertensive drug and the occurrence of AA/AAG will be based on clinical judgment. To achieve this, other potential causes will be considered and studied, including the natural progression of the underlying diseases, concurrent treatments, other risk factors, and the temporal correlation of the event with the investigational product. The technical data sheet of the drug will serve as the reference safety information for this study.

Related adverse event: The temporal correlation between AE and the antihypertensive drug indicates a possible causal relationship, which cannot be explained by factors such as the patient's clinical status or therapeutic interventions.

Unrelated adverse event: The temporal relationship between the adverse event (AE) and the antihypertensive drug suggests an unlikely causal connection. Alternatively, other factors (medications or concurrent conditions) and additional therapeutic interventions may offer a satisfactory explanation for the AE.

b) Information on adverse events

Detection method: Adverse events will be recorded from the informed consent stage and at each visit through careful clinical observation, patient communication, and open questioning by the investigator.

Record of adverse events: All AEs and AAEs not mentioned in the data sheet must be documented in the clinical history and CRD. This documentation will include start and end dates, event descriptions, severity, evolution, outcome, relationship with the antihypertensive drug, and measures taken. At each visit, all AEs that the patient has experienced since the previous visit must be documented on the Adverse Events Form, provided they are not already included in the technical file. If an adverse event was pre-existing and has worsened in terms of intensity or frequency, the direction of the change should be indicated.

c) Procedures for immediate notification of serious or unexpected adverse events

Prompt reporting of serious adverse events by the investigator

In the event of an SAE, the investigator will inform the pharmacovigilance unit within 24 hours of becoming aware of it.

The initial communication of the SAAs must be in writing using the provided Serious Adverse Events Notification Form provided by the Unit responsible for the pharmacovigilance of the trial (**ANNEX 1**). The form must be sent via email to: uicec@incliva.es.

The person in charge of Pharmacovigilance will review the received form and, if necessary, request additional information from the investigator. The investigator will provide this information when requested and, if there are changes in their initial assessment of severity or causation, will communicate these changes. The notification procedure described earlier will be followed for conveying monitoring information. The person in charge of Pharmacovigilance will maintain a detailed record of all SAEs or significant events communicated by researchers.

Deaths of subjects participating in the study must be notified using the notification form within 24 hours of knowledge of the death, as long as the subject is within the follow-up period.

Serious adverse events that do not require immediate notification by the investigator

The following serious adverse events do not require immediate notification by form:

- Thromboembolic events (deep vein thrombosis, pulmonary thromboembolism)
- Neurological events (stroke, cerebrovascular accident, acute confusional syndrome)
- Cardiological events (angina, myocardial infarction, cardiac arrhythmia, decompensation of heart failure, hypotensive crises)
- Impaired renal function
- Gastrointestinal bleeding
- Need for blood transfusion
- Hospitalization due to hydroelectrolyte disorder

In any case, there will be a section in the CRD to collect information on possible serious adverse events reported by the patient during follow-up visits. Serious adverse events that do not require immediate notification must be included in the clinical history and in the CRD, and will be included in the periodic safety report.

On the other hand, the study will collect data related to urgent hospital admissions due to episodes of angina, myocardial infarction, stroke, and hypotensive crises, as well as death from any cause, along with outpatient and emergency visits due to hypotensive crises, as part of the tertiary objectives of the study. Given the characteristics of the included population (and the results of the TASMIN-SR[17] study), figures of less than 3% are expected for the combined endpoints; therefore, it is not anticipated that differences will be detectable. For these serious events, as well as for deaths, the Family Physician Investigators responsible for the patients (MEFI) will review the medical history and determine their potential relationship with the intervention. Previous trials have failed to find any evidence of such a relationship.

Expedited notification of serious and unexpected adverse reactions

The investigator will notify the Sponsor of all suspected serious and unexpected adverse reactions (SARGI), who will be responsible for reporting them in accordance with current regulations on clinical trials to the AEMPS (and authorities of the Autonomous Communities, if

applicable) within a maximum period of 15 calendar days from the time they become known. When the SARGI has resulted in the patient's death or endangered their life, the notification will be made within a maximum period of seven calendar days from the time it becomes known. Subsequent event-related information will be provided within eight days.

d) Pregnancy

As the study includes individuals over 40 years of age, there is a possibility of pregnancy during the follow-up period. However, due to the naturalistic conditions of the study, it is not deemed necessary to enforce contraceptive measures for women of childbearing age (which could additionally introduce added risks). While the ADAMPA protocol does not assign drugs (and therefore there are no risks of adverse events associated with drug treatments derived from the study, as any such risks would arise from drugs prescribed by physicians independent of the study), pregnancy can complicate the management of hypertension (and vice versa) and necessitate specific and distinct monitoring beyond that outlined in the intervention protocol. Consequently, should a pregnancy occur during the follow-up period, affected women will be excluded from the assigned protocol and will be managed freely by their doctor(s), while maintaining endpoint follow-up and inclusion in the intention-to-treat analysis.
No additional explorations, tests, or complementary evaluations will be conducted beyond those undertaken in routine clinical practice.

e) Periodic safety report

During the study, the Unit responsible for Pharmacovigilance will prepare annual safety reports in line with recommendations from the ICH E2F guide. These reports will be presented to regulatory authorities and the CEIm following the established calendar in current legislation.

11. Statistical analysis

11.1 Sample size

A sample size of 382 patients was estimated in order to have 90% power to detect a 5 mmHg (SD 15 mmHg) difference in SBP between groups (primary outcome) with a two-tailed contrast and an alpha error of 0.05. This figure represents a clinically relevant difference based on previous trials.[15,16] We increased this figure to 458 participants (20% increase) to account for possible dropouts and loss to follow-up.

11.2 Statistical analysis

Analysis will be on an intention-to-treat basis for complete cases. We will use mixed models (general linear modeling –GLM) to compare SBP at 12 months between the intervention and control groups. This analysis will be presented in both crude and adjusted forms for the different covariates of interest (baseline BP, gender, GP/PCC-random effect, diabetes, etc.). A sensitivity analysis will be performed to examine the potential effect of missing data, which will include substitution by multiple imputation, replacement of data lost by the most recent data or by the mean of the series. Additionally, analyses of the main outcome measure by subgroups of age, gender, comorbidity, level of chronicity, better control at baseline, etc. will be performed.

Differences in secondary outcome measures (DBP, percentage of patients controlled, PDC, persistence and TI) will be analyzed using methods similar to those used for analysis of the main outcome measure.

Economic analysis

The economic analysis will include a cost-consequence analysis, estimating both the costs (hospitalizations, outpatient visits, emergency visits and antihypertensive drugs) and the potential benefits (e.g. reduced incidence of stroke, myocardial infarction, etc.) in natural units. In addition, we will collect information on Health-Related Quality of Life (HRQOL) through the EQ-5d questionnaire, which will allow us to obtain utilities and therefore perform a cost-utility analysis with the estimated benefits in terms of Quality-Adjusted Life-Years (QALY).

A modeling will be performed to obtain longer-term predictions of the results observed in the trial. The results on which this modeling will be based will be survival, quality of life and costs associated with clinical events. A sensitivity analysis (deterministic and probabilistic) will be performed to analyze the robustness of the results. Key parameters will be modified to determine their impact on results. All analyses will be performed using STATA version 14.

Quality Sub-study

Qualitative research techniques will seek to provide an in-depth understanding of the positive elements and areas of improvement related to self-titration and self-monitoring intervention. To this end, two meetings will be held, one with professionals (GPs and nurses) and one with patients, using the Nominal Group Technique (NGT). The NGT is a working methodology that establishes a framework for highly structured interaction that enables participation and equal consideration of the contributions of all members of the working group, and allows the identification of priorities, consensus and disagreement, solution generation and decision-making in an agile and objective manner (24).

12. ETHICAL ASPECTS

12.1 Ethics Committee (Helsinki) and Authorities

The investigator must conduct the study in accordance with the principles of the Declaration of Helsinki, the Good Clinical Practice (GCP) standards, as described in the Harmonized Tripartite Guide to Good Clinical Practice (International Conference on Harmonization, ICH, 1996). and Royal Decree 1090/2015, of December 4, which regulates clinical trials with drugs, the Ethics Committees for Research with drugs and the Spanish Registry of Clinical Studies.

The study protocol and its attached documentation will be sent for approval to the clinical research ethics committee (CEIC-HCUV) as well as to the Spanish Agency for Medicines and Health Products (AEMPS).

12.2 Informed consent

Participant informed consent (IC) will be mandatory for study inclusion. The IC will be presented to participants during the selection session by the MEFI. This document will detail the trial's nature, patient implications for participating, protocol conditions, and potential risks associated with participation. The IC will include agreement to participate and permission to access clinical history for information on medication, medical history, clinical outcomes, and resource utilization. Participants will retain the right to leave the study at any time without compromising future healthcare.

Participants will have ample time to review information, consult the MEFI or relevant individuals, and make an informed decision. The written IC will include participant signature

and date, as well as date and signature of the person obtaining consent. The original IC will be kept in the investigator's file, with copies given to the patient and the MIC team.

12.3 Data protection

Strict compliance with Law 15/1999 of December 13 on the Protection of Personal Data and its associated regulations is guaranteed.

Confidentiality of each patient's data will be maintained at all times. The original data will be retained at the health center and may only be accessed by study researchers, individuals responsible for monitoring, the Clinical Research Ethics Committee, and Spanish Health Authorities in case of inspection. Data used in the study will be identified by a code. The researcher is the only individual authorized to and responsible for knowing the source of the collected data and connecting them to the respective patient.

The main researchers and collaborators participating in the study, when handling patient data, will take appropriate measures to safeguard the data and prevent unauthorized third-party access.

12.4 Monitoring and auditing

Platform for Clinical Research Units and Clinical Trials Spanish Clinical Research Network (SCReN) will monitor the study.

During the study, the monitor will visit the participating centers to verify that the patient documents are complete, that the data entry in the Data Collection Notebook (CRD) is accurate, that the study protocol and the standards of Good Clinical Practice and that progress in recruitment is correct.

The study principal investigator and collaborators should be available to assist the monitor during these visits.

The investigator must keep all the source documentation of each patient included in the study (hospital medical records, including medical and demographic information and the results of any other test or evaluation). All the information that is registered in the Data Collection Notebook (CRD) must be verifiable with the source documents of the patient's file. The investigator must retain the original informed consent signed by the patient (a signed copy will be provided to the patient).

Monitoring will require full verification of the existence of informed consent, of compliance with the inclusion/exclusion criteria, and of the data to be used for all primary endpoints.

Additional checks will be carried out to verify that the source data matches the data in the Data Collection Notebook (CRD) according to the study monitoring plan.

The ICH Harmonized Tripartite Standards for Good Clinical Practice state that research sites and all data, including source data, should be available for inspection by competent authorities. The Patient Information Sheet (HIP) will inform patients that their medical records may be reviewed during these audits.

12.5 Protocol Compliance.

This protocol defines the objectives and procedures of the study and the data to be collected from study participants. An investigator may not, under any circumstances, collect additional data or carry out any other procedure for any other purpose not described in the study protocol.

Investigators must exercise caution to prevent deviations from the protocol. If an investigator believes that a deviation would enhance the study's execution, it must be regarded as an amendment to the protocol. This amendment cannot be implemented until approved by the study investigators, the Clinical Research Ethics Committee (CEIm), and, when necessary, the Spanish Agency of Medicines and Medical Devices (AEMPS).

12.6 Early Termination or Suspension of the Study

The study will be interrupted when statistically significant differences are observed in the presence of serious adverse events related to the intervention.

13. FUNDING AND INSURANCE

13.1 Funding

Granted concurrent research aid from the National R+D+i Plan (FIS Projects) in its last call (PI16/02130).

13.2 Insurance

In accordance with the provisions of articles 2.j) and 9.4 of Royal Decree 1090/2015, of December 4, which regulates clinical trials with drugs, the Research Ethics Committees with drugs and the Spanish Registry of Clinical Studies, damages to the subject of the study that could result as a consequence of a low level intervention clinical trial will not need to be covered by an insurance contract or financial guarantee if they are covered by civil liability insurance. individual or collective professional or equivalent financial guarantee from the health center where the clinical trial is carried out. In the case of the Valencian Community, damages to the subject of the study that could result as a result of a clinical trial with a low level of intervention are covered by the civil liability insurance subscribed by the Ministry of Universal Health and Public Health.

14. PUBLICATION POLICY

The inclusion as co-authors of the study researchers will be considered according to their participation in the different stages of the study and the preparation of the manuscripts. All of them will have access to the drafts of the manuscripts prior to their submission to the different scientific journals.

14.1 Publication of the study protocol and results

The main design elements of this protocol will be published in a publicly accessible database (Spanish Registry of Clinical Studies). In addition, upon completion of the study and its reports, the results of this study will be submitted for publication or become part of a publicly accessible database of clinical trial results (as appropriate).

15. FINAL REPORT

A final report will be sent, after the completion of the study, to the AEMPS and the competent bodies of the autonomous communities where it is carried out, within the period indicated according to the requirements of current regulations.

15. REFERENCES

1. Murphy T, Naghavi M, Neal B, Lim SS, Vos T, Flaxman AD et al. A comparative risk assessment of burden of disease and injury attributable to 67 risk factors and risk factor clusters in 21 regions, 1990-2010: a systematic analysis for the Global Burden of Disease Study 2010. Lancet. 2012;380(9859):2224-60
2. Catalá-López F, Sanfélix-Gimeno G, García-Torres C, Ridao M, Peiró S. Control of arterial hypertension in Spain: a systematic review and meta-analysis of 76 epidemiological studies on 341632 participants. J Hypertens. 2012;30(1):168-76.
3. Catalá-López F, Ridao M, Sanfélix-Gimeno G, Peiró S. Trends of uncontrolled blood pressure in Spain: an updated meta-regression analysis. J Hypertens. 2013;31(3):630-1.
4. Joffres M, Falaschetti E, Gillespie C, Robitaille C, Loustalot F, Poulter N, et al. Hypertension prevalence, awareness, treatment and control in national surveys from England, the USA and Canada, and correlation with stroke and ischaemic heart disease mortality: a cross-sectional study. BMJ Open. 2013;3(8):e003423.
5. Redon J, Olsen MH, Cooper RS, Zurriaga O, Martinez-Beneito MA, Laurent S, et al. Stroke mortality and trends from 1990 to 2006 in 39 countries from Europe and Central Asia: implications for control of high blood pressure. Eur Heart J. 2011;32(11):1424-31.
6. Zhao Y, Yan H, Marshall RJ, Dang S, Yang R, Li Q, et al. Trends in population blood pressure and prevalence, awareness, treatment, and control of hypertension among middle-aged and older adults in a rural area of Northwest China from 1982 to 2010. PLoSOne. 2013;8(4):e61779.
7. James PA, Oparil S, Carter BL, Cushman WC, Dennison-Himmelfarb C, Handler J, et al. 2014 evidence-based guideline for the management of high blood pressure in adults: report from the panel members appointed to the Eighth Joint National Committee (JNC 8). JAMA. 2014;311 (5):507–20.
8. Mancia G, Fagard R, Narkiewicz K, Redón J, Zanchetti, Böhm M et al. 2013 ESH/ESC Guidelines for the management of arterial hypertension. The Task Force for the management of arterial hypertension of the European Society of Hypertension (ESH) and of the European Society of Cardiology (ESC). J Hypertension. 2013; 31:1281–1357
9. Navar-Boggan AM, Pencina MJ, Williams K, Sniderman AD, Peterson ED. Proportion of US adults potentially affected by the 2014 hypertension guideline. JAMA. 2014;311(14):1424-9.
10. McManus RJ, Wood S, Bray EP, Glasziou P, Hayen A, Heneghan C, et al. Self-monitoring in hypertension: a web-based survey of primary care physicians. J Hum Hypertens. 2014;28(2):123-7.
11. Logan AG, Dunai A, McIsaac WJ, Irvine MJ, Tisler A. Attitudes of primary care physicians and their patients about home blood pressure monitoring in Ontario. J Hypertens. 2008;26(3):446-52.
12. Dalfó i Baqué A, Capillas Peréz R, Guarch Rocarias M, Figueras Sabater M, Ylla-Català Passola A, Balañá Vilanova M, et al. Effectiveness of self-measurement of blood pressure in patients with hypertension: the Dioampa study. Aten Primaria. 2005;35(5):233-7.
13. Marquéz Contreras E, Martin de Pablos JL, Espinosa García J, Casado Martínez JJ, Sánchez López E, Escribano J. Grupo de Trabajo sobre Cumplimiento de la Asociación de la Sociedad Española de Hipertensión Arterial y Liga Española par la Lucha contra la Hipertensión Arterial (SEH-LELHA). Prevention of therapeutic inertia in the treatment of arterial

hypertension by using a program of home blood pressure monitoring. Aten Primaria.2005;44(2):89-96.

14. Glynn LG, Murphy AW, Smith SM, Schroeder K, Fahey T. Interventions used to improve control of blood pressure in patients with hypertension. Cochrane Database Syst Rev. 2010;(3):CD005182.
15. Uhlig K, Patel K, Ip S, Kitsios GD, Balk EM. Self-measured blood pressure monitoring in the management of hypertension: a systematic review and meta-analysis. Ann Intern Med. 2013;159(3):185-94.
16. McManus RJ, Mant J, Bray EP, Holder R, Jones MI, Greenfield S, et al. Telemonitoring and self-management in the control of hypertension (TASMINH2): a randomised controlled trial. Lancet. 2010;376(9736):163-72.
17. McManus RJ, Mant J, Haque MS, Bray EP, Bryan S, Greenfield SM, et al. Effect of self-monitoring and medication self-titration on systolic blood pressure in hypertensive patients at high risk of cardiovascular disease: the TASMIN-SR randomized clinical trial. JAMA. 2014;312(8):799-808.
18. Margolius D, Bodenheimer T, Bennett H, Wong J, Ngo V, Padilla G, et al. Health coaching to improve hypertension treatment in a low-income, minority population. Ann Fam Med. 2012;10:199-205.
19. Delbecq A, Van De Ven A. A Group Process Model For Problem Identification and Program Planning. The Journal of Applied Behavioral Science. 1971;7(4):466-492.

Unión Europea<br>Fondo Europeo de Desarrollo Regional<br>"Una manera de hacer Europa"<br>SCReN Spanish Clinical Research Network	**SERIOUS ADVERSE EVENT REPORTING FORM**	**Protocol code: ADAMPA**
	**Case number:** ______________________________<br>**Notification number:** ___________________________	**N EudraCT: 2016-003986-25**

Subject code: _______________	TYPE OF REPORT: ☐ Initial ☐ Follow-up

1. CENTER INFORMATION

Center code:	Principal Investigator:	
Person reporting the adverse event:		Tel:
Fax:	Mail:	

2. INFORMACIÓN DEL SUJETO

Sex	Age (at the begining of AE)	Grupo de edad (rellenar sólo si se desconoce la edad del sujeto)	Birth date (dd-mmm-yyyy)	Weight (kg)	Height (cm)
☐ Male<br>☐ Female	_________<br>☐ Years ☐ Months ☐ Days	☐ Neonate ☐ Infant<br>☐ Child ☐ Adolescent<br>☐ Adult ☐ Elderly	_ _ – _ _ _ – _ _ _ _		

3. ADVERSE EVENT

Serious adverse event (Specify the diagnosis or syndrome, if known. If unknown include signs and symptoms.)**:**

……

……

DESCRIPTION OF THE ADVERSE EVENT (Provide all the information about the circumstances, the sequence, the diagnosis and the treatment of the AE)

Start date (dd-mmm-yyyy): _ _ – _ _ _ – _ _ _ _   End date: (dd-mmm-yyyy): _ _ – _ _ _ – _ _ _ _

Severity criterion:	Outcome (status at the time of notification):
☐ Death	☐ Recovered
☐ Life threatening	☐ In recovery
☐ Requires or prolongs hospitalization	☐ Not recovered
☐ Permanent or significant disability	☐ Recovered with sequelae
☐ Birth defect or congenital anomaly	☐ Lethal
☐ Clinically relevant	☐ Unknown

In case of death, fill in the following information:

Date of death: _ _ – _ _ _ – _ _ _ _   Cause: ____

Was an autopsy performed: ☐ Yes ☐ No ☐ Unknown

Indicate the cause of death according to the autopsy: ____________________________________

	**SERIOUS ADVERSE EVENT REPORTING FORM**	**Notification number:** ________________
	**Protocol code: ADAMPA**	**Patient number :** ___________________

4. INVESTIGATIONAL PRODUCT/DRUG (Indicate the treatment(s) assigned to the subject after randomization)
If you need more space, please use copies of this page and check this box ☐

Medication	Daily dose (units)	Frecuency	Route	Start date (dd-mmm-yyyy)	End date (dd-mmm-yyyy) (if it continues check the box)	Causal relationship
					☐	☐ Related<br>☐ Not related
					☐	☐ Related<br>☐ Not related
					☐	☐ Related<br>☐ Not related

Action taken with medication in response to AE	☐ Medication withdrawal ☐ Dose decrease ☐ No changes<br>☐ Temporal interruption of medication ☐ Dose increase ☐ Not applicable<br>☐ Unknown
Did the AE subside when the medication was stopped or the dose was reduced?	☐ Yes ☐ No ☐ Unknown ☐ Not applicable
Did the AE reappear when the medication was reintroduced?	☐ Yes ☐ No ☐ Unknown ☐ Not applicable

5. CONCOMITANT MEDICATION (Include those concomitant and basic treatments that you have taken in the two weeks prior to the AA start date. Do not include the treatment administered to treat the AA or that administered after the AE start date)
If you need more space, please use copies of this page and check this box ☐

Medication	Daily dose (units)	Frecuency	Route	Start date (dd-mm-yyyy)	End date (dd-mmm-yyyy) (if it continues check the box)	Causal relationship	Indication
					☐	☐ Related<br>☐ Not related	
					☐	☐ Related<br>☐ Not related	
					☐	☐ Related<br>☐ Not related	

Action taken with medication in response to AE	☐ Medication withdrawal ☐ Dose decrease ☐ No changes<br>☐ Temporal interruption of medication ☐ Dose increase ☐ Not applicable<br>☐ Unknown
Did the AE subside when the medication was stopped or the dose was reduced?	☐ Yes ☐ No ☐ Unknown ☐ Not applicable
Did the AE reappear when the medication was reintroduced?	☐ Yes ☐ No ☐ Unknown ☐ Not applicable

6. ALTERNATIVE CAUSE

Is there any possibility that the AE is related to a cause other than medication?
☐ Yes ☐ No If so, specify (extend information in the Serious adverse event description section if necessary):
____
____

7. RELEVANT MEDICAL HISTORY
If you need more space, please use copies of this page and check this box ☐

Pathological history	Start date (dd-mmm-yyyy)	End date (dd-mmm-yyyy)	(if it continues check the box)
			☐
			☐
			☐
			☐

Complementary information of the data of the Medical History

8. LABORATORY DATA AND OTHER COMPLEMENTARY EXAMINATIONS: (indicate only the relevant test results to document the reported serious adverse event)
If you need more space, please use copies of this page and check this box ☐

TEST	TEST DATE (dd-mmm-yyyy)	RESULT (Units)	Range reference	OBSERVATIONS

Complementary information on laboratory data and other examinations:

Signature of the reporting researcher	Date of notification
**Signature of the Pharmacovigilance Department Head**	**Date of notification reception**

SEND IMMEDIATELY BY EMAIL TO THE PHARMACOVIGILANCE DEPARTMENT OF INCLIVA

uicec@incliva.es
