# Supplementary material for: Long-Term Effect of Home Blood Pressure Self-Monitoring Plus Medication Self-Titration for Patients With Hypertension: A Secondary Analysis of the ADAMPA Randomized Clinical Trial
Source: JAMA Netw Open. 2024 May 10;7(5):e2410063. doi: 10.1001/jamanetworkopen.2024.10063 (PMC11087839; doi:10.1001/jamanetworkopen.2024.10063)
Supplement: Supplement 2. — eAppendix 1. Exclusion Criteria eAppendix 2. HBPM and Self-Titration Instructions Sheets eAppendix 3. Individualized Adjustment Plan Sheet eTable 1. Baseline Characteristics of the ADAMPA Trial Patients Completing the 24-Month Visit and Patients Not Completing the 24-Month Visit eTable 2. Baseline Characteristics of the ADAMPA Trial Patients Completing the 24-Month Visit and Patients Not Completing the 24-Month Visit by Study Group eTable 3. Home Blood Pressure Monitoring and Medication Changes at 24 Months eTable 4. Self-Titration of Antihypertensive Medication in the Intervention Group (n=111) During the Whole Study Period and the Extension Phase eTable 5. Differences in Systolic and Diastolic Blood Pressure Among Study Groups at 24 Months, Crude And Adjusted: Sensitivity Analysis Including All Patients Attending the 12-Month Visit (n=312) Using Multiple Imputation eTable 6. Differences in Systolic and Diastolic Blood Pressure Among Study Groups at 24 Months, Crude and Adjusted: Sensitivity Analysis Including All Patients Attending the 12-Month Visit (n=312) Using the Last Observation Carried Forward eTable 7. Number and Percentage of Patients Achieving Their Target BP at the Final Follow-Up Visit in the Intervention and Control Groups eTable 8. Adverse Events During the Extension Follow-Up Period eTable 9. Behavioural Risks and Health-Related Quality of Life at 12 Months and at the Final Follow-Up Visit eTable 10. Health Care Services Utilization During the Extension Follow-Up Period eFigure. Mean SBP and DBP in Follow-Up Visits at Baseline, 6, 12 Months and the Final Follow-Up Visit for the Intervention (n=111) and Control Groups (n=108) [file jamanetwopen-e2410063-s002.pdf]

Supplementary Online Content

Martínez-Ibáñez P, Marco-Moreno I, García-Sempere A, et al; ADAMPA Research Group. Long-term effect of home blood pressure self-monitoring plus medication self-titration for patients with hypertension: a secondary analysis of the ADAMPA randomized clinical trial. *JAMA Netw Open.* 2024;7(5):e2410063. doi:10.1001/jamanetworkopen.2024.10063

eAppendix 1. Exclusion Criteria
eAppendix 2. HBPM and Self-Titration Instructions Sheets
eAppendix 3. Individualized Adjustment Plan Sheet
eTable 1. Baseline Characteristics of the ADAMPA Trial Patients Completing the 24-Month Visit and Patients Not Completing the 24-Month Visit
eTable 2. Baseline Characteristics of the ADAMPA Trial Patients Completing the 24-Month Visit and Patients Not Completing the 24-Month Visit by Study Group
eTable 3. Home Blood Pressure Monitoring and Medication Changes at 24 Months
eTable 4. Self-Titration of Antihypertensive Medication in the Intervention Group (n=111) During the Whole Study Period and the Extension Phase
eTable 5. Differences in Systolic and Diastolic Blood Pressure Among Study Groups at 24 Months, Crude And Adjusted: Sensitivity Analysis Including All Patients Attending the 12-Month Visit (n=312) Using Multiple Imputation
eTable 6. Differences in Systolic and Diastolic Blood Pressure Among Study Groups at 24 Months, Crude and Adjusted: Sensitivity Analysis Including All Patients Attending the 12-Month Visit (n=312) Using the Last Observation Carried Forward
eTable 7. Number and Percentage of Patients Achieving Their Target BP at the Final Follow-Up Visit in the Intervention and Control Groups
eTable 8. Adverse Events During the Extension Follow-Up Period
eTable 9. Behavioural Risks and Health-Related Quality of Life at 12 Months and at the Final Follow-Up Visit
eTable 10. Health Care Services Utilization During the Extension Follow-Up Period
eFigure. Mean SBP and DBP in Follow-Up Visits at Baseline, 6, 12 Months and the Final Follow-Up Visit for the Intervention (n=111) and Control Groups (n=108)

This supplementary material has been provided by the authors to give readers additional information about their work.

© 2024 Martínez-Ibáñez P et al. *JAMA Network Open.*

**eAppendix 1.** Exclusion Criteria
Inability to self-manage their BP, including dementia or significant cognitive impairment (as assessed by the researcher performing the recruitment)
A history of orthostatic hypotension (fall> 20 mmHg from SBP after taking the orthostatic position)
SBP > 200 or DBP > 100 mmHg in the baseline examination
Being prescribed more than 4 antihypertensive drugs
Inclusion in another hypertension study or clinical trial
Presence of tremor or neurological disease that makes it difficult to perform HBPM
Presence of arrhythmia
Presence of terminal illness
Chronic incapacitation to leave home
Acute cardiovascular event in the 3 months previous to the baseline visit
Hypertension managed directly by other specialist doctors outside the primary care environment
Pregnant women or those actively seeking to become pregnant
Having a household member already enrolled in the study
Non-residents or temporary residents
Abreviations: BP: blood pressure; SBP: systolic blood pressure; DBP: diastolic blood pressure; HBPM: Home blood pressure monitoring.

© 2024 Martínez-Ibáñez P et al. *JAMA Network Open.*

eAppendix 2. HBPM and Self-Titration Instructions Sheets

HOW TO ACT ACCORDING TO YOUR BLOOD PRESSURE MEASUREMENTS

Remember:

- Take your blood pressure twice a day, once in the morning and once in the evening, FOR the first seven days of each month.
- Whenever your BP is taken, you should take it twice, waiting for 1-2 minutes in between.
- Write down the lowest measure of both systolic blood pressure (TOP reading) measurements in your monthly notebook.
- In the case of a strange value, or a possible error in a measurement, repeat it to get 2 valid measurements. Once you have entered your blood pressure values in your monthly notebook, act according to the following TABLE OF COLOURS OF ACTION, **unless your doctor has indicated otherwise**.

BLOOD PRESSURE VALUES (morning or afternoon)		READING	ACTION
**VERY HIGH**	Your systolic blood pressure (TOP reading) is **180 or more** or Your diastolic blood pressure (BOTTOM reading) is **100 or more**	**Your blood pressure is too high**	**Contact your doctor or visit your health care centre.**
**HIGH**	If your systolic blood pressure (TOP reading) or your diastolic blood pressure (BOTTOM reading) is above the target values marked by your doctor.	**Your blood pressure is raised**<br><br>If **FOUR** or more readings a week, once a month were high, proceed with the medication change instructions.	**1st step: Follow instructions provided by your doctor.**<br><br>**2nd step: make an appointment with your doctor within 3 weeks after self-adjustment.**
**NORMAL**	Your blood pressure values are in the range of your BP target.	Your blood pressure is well controlled	Continue with your usual medication and when you go to visit your doctor for any reason, show your doctor your blood pressure booklet measurements.
**LOW**	Your systolic blood pressure (TOP reading) is **100 or less.**	**Your blood pressure is too low**	**Contact your doctor or visit your health care centre.**

RED: Contact your doctor or visit your health centre.
YELLOW: Proceed to self-adjustment at home with your doctor's instructions and schedule an appointment for 3 weeks after self-adjustment.

IN CASE OF DOUBT, CONTACT YOUR GENERAL PRACTITIONER.

Adapted and modified from: The Colour Coding Chart. Supplementary webappendix in: McManus RJ, Mant J, Bray EP, et al. Telemonitoring and self-management in the control of hypertension (TASMINH2): a randomised controlledtrial. Lancet 2010; published online July 8. DOI:10.1016/S0140-6736(10)60964-6.

© 2024 Martínez-Ibáñez P et al. *JAMA Network Open.*

eAppendix 3. Individualized Adjustment Plan Sheet

INCLIVA | VLC
Instituto de Investigación Sanitaria

SCReN Spanish Clinical Research Network ISCIII

ADAMPA STUDY

ADJUSTMENT SHEET ANTIHYPERTENSIVE MEDICATION

NAME AND SURNAME OF PATIENT: ____

TARGET BLOOD PRESSURE: ________/________

If your Systolic Pressure (the HIGH) or your Diastolic Pressure (the LOW), morning or afternoon, are above your TARGET PRESSURE set by your doctor, 4 days or more out of the first 7 days of the month, proceed as follows:

....

....

....

Do not forget to request a consultation with your doctor 3 weeks after adjusting the medication. If you have any questions, consult your family doctor.

Signed ....

Family doctor's name. ....

© 2024 Martínez-Ibáñez P et al. *JAMA Network Open.*

eTable 1. Baseline Characteristics of the ADAMPA Trial Patients Completing the 24-Month Visit and Patients Not Completing the 24-Month Visit

	No. (%)		
	**Patients completing the 24-month visit (n=219)**	**Patients not completing the 24-month visit (n=93)**	**p value[a]**
Female	120 (54.8)	46 (49.5)	0.46
Male	99 (45.2)	47 (50.5)	
Age, years, mean (SD)	64.3 (10.1)	64.5 (9.9)	0.84
Systolic blood pressure, mmHg, mean (SD)	155.6 (13.1)	154.3 (12.4)	0.4
Diastolic blood pressure, mmHg, mean (SD)	90.8 (7.7)	88.6 (8.6)	0.03
Body mass index			
Normal (18-24 kg/m^2)	35 (16.0)	12 (12.9)	
Overweight (25-30 kg/m^2)	90 (41.1)	43 (46.2)	0.73
Obese (≥30 kg/m^2)	93 (42.5)	38 (40.9)	
Body mass index, mean (SD)	29.8 (5.0)	29.9 (4.9)	0.8
Level of education			
No qualification	12 (5.5)	8 (8.6)	
Primary education	96 (43.8)	32 (34.4)	0.4
Secondary education	69 (31.5)	34 (36.6)	
University degree or higher	42 (19.2)	19 (20.4)	
Marital status			
Single	16 (7.3)	5 (5.4)	
Married	149 (68.0)	62 (66.7)	0.84
Divorced	13 (5.9)	9 (9.7)	
Widowed	34 (15.5)	13 (14.0)	
Employment status			
Permanent work	67 (30.6)	26 (28.0)	
Temporary work	1 (0.5)	5 (5.4)	
Housewife	32 (14.6)	4 (4.3)	<0.001
Unemployed	14 (6.4)	4 (4.3)	
Pensioner	105 (48.0)	54 (58.1)	
Smoking	47 (21.5)	17 (18.3)	0.63
Sedentarism	95 (43.4)	43 (46.2)	0.73
HRQoL (EQ5D), mean (SD)	0.8 (0.2)	0.9 (0.2)	0.28
Comorbidities			

© 2024 Martínez-Ibáñez P et al. *JAMA Network Open.*

Diabetes	52 (23.7)	23 (24.7)	0.97
Cerebrovascular disease	7 (3.2)	3 (3.2)	1.00
Angina	2 (0.9)	0 (0.0)	0.98
Acute myocardial infarction	4 (1.8)	0 (0.0)	0.33
Peripheral artery disease	5 (2.3)	0 (0.0)	0.63
Chronic kidney disease	15 (6.9)	3 (3.2)	0.75
Years of onset hypertension, mean (SD)	10.8 (9.0)	11.6 (10.0)	0.73
N antihypertensive drugs, mean (SD)	1.7 (0.9)	1.7 (1.0)	0.15
N concomitant treatments, mean (SD)	2.9 (2.3)	2.9 (2.0)	0.17
Home blood pressure monitoring	67 (30.6)	20 (21.5)	0.39

Abbreviations: HRQoL: health-related quality of life; EQ5D: EuroQol-5D.
[a] Significance level of the $\chi 2$ test of Independence among randomized groups for binary and categorical variables or the 1-way analysis of variance among randomized groups for continuous variables. $P < .05$ is statistically significant.

© 2024 Martínez-Ibáñez P et al. *JAMA Network Open.*

eTable 2. Baseline Characteristics of the ADAMPA Trial Patients Completing the 24-Month Visit and Patients Not Completing the 24-Month Visit by Study Group

	**No. (%)**			
	**Patients completing the 24-month visit (n=219)**		**Patients not completing the 24-month visit (n=93)**	
	**Intervention (n=111)**	**Control (n=108)**	**Intervention (n=45)**	**Control (n=48)**
Female	61 (54.9)	59 (54.6)	25 (54.6)	21 (43.7)
Male	50 (45.1)	49 (45.4)	20 (44.4)	27 (56.3)
Age, years, mean (SD)	65.1 (9.57)	63.4 (10.50)	64.1 (9.9)	64.9 (9.9)
Systolic blood pressure, mmHg, mean (SD)	155.3 (13.2)	156.0 (13.2)	154.9 (12.9)	153.6 (12.1)
Diastolic blood pressure, mmHg, mean (SD)[a]	90.1 (7.9)	91.5 (7.4)	88.6 (8.1)	88.6 (9.1)
Body mass index				
Normal (18-24 kg/m^2)	18 (16.2)	17 (15.7)	7 (15.6)	5 (10.4)
Overweight (25-30 kg/m^2)	47 (42.3)	43 (39.8)	16 (35.6)	27 (56.3)
Obese (≥30 kg/m^2)	45 (40.5)	48 (44.4)	22 (48.9)	16 (33.3)
Body mass index, mean (SD)	29.6 (4.9)	29.9 (4.9)	30.9 (5.6)	29.0 (4.0)
Level of education				
No qualification	7 (6.3)	5 (4.6)	6 (13.3)	2 (4.2)
Primary education	50 (45.1)	46 (42.6)	14 (31.1)	18 (37.5)
Secondary education	33 (29.7)	36 (33.3)	18 (40.0)	16 (33.3)
University degree or higher	21 (18.9)	21 (19.4)	7 (15.6)	12 (25.0)
Marital status				
Single	9 (8.1)	7 (6.5)	0 (0.0)	5 (10.4)
Married	75 (67.6)	77 (71.3)	29 (64.4)	33 (68.8)
Divorced	10 (9.01)	7 (6.5)	7 (15.6)	2 (4.2)
Widowed	17 (15.3)	17 (15.7)	7 (15.6)	6 (12.5)
Employment status[b]				
Permanent work	31 (27.9)	36 (33.3)	12 (26.7)	14 (29.2)
Temporary work	0 (0.0)	1 (0.9)	3 (6.7)	2 (4.2)
Housewife	16 (14.4)	16 (14.8)	1 (2.2)	3 (6.3)
Unemployed	10 (9.0)	4 (3.7)	4 (8.9)	0 (0.0)
Pensioner	54 (48.7)	51 (47.2)	25 (55.6)	29 (60.4)
Smoking	20 (18.0)	27 (25.0)	8 (17.8)	9 (18.8)
Sedentarism	95 (43.4)	43 (46.2)	25 (55.6)	18 (37.5)
HRQoL (EQ5D), mean (SD)	0.8 (0.2)	0.9 (0.2)	0.9 (0.2)	0.9 (0.2)
Comorbidities				

© 2024 Martínez-Ibáñez P et al. *JAMA Network Open.*

Diabetes	52 (23.7)	23 (24.7)	12 (26.7)	11 (22.9)
Cerebrovascular disease	7 (3.2)	3 (3.2)	1 (2.2)	2 (4.2)
Angina	2 (0.9)	0 (0.0)	0 (0.0)	0 (0.0)
Acute myocardial infarction	4 (1.8)	0 (0.0)	0 (0.0)	0 (0.0)
Peripheral artery disease	5 (2.3)	0 (0.0)	0 (0.0)	0 (0.0)
Chronic kidney disease	15 (6.9)	3 (3.2)	2 (4.4)	1 (2.1)
Years of onset hypertension, mean (SD)	10.8 (9.0)	11.6 (10.0)	11.27(10.0)	11.9 (10.1)
N antihypertensive drugs, mean (SD)	1.7 (0.9)	1.7 (1.0)	1.6 (1.0)	1.9 (0.9)
N concomitant treatments, mean (SD)	2.9 (2.3)	2.9 (2.0)	2.5 (1.9)	3.2 (2.1)
Home blood pressure monitoring	67 (30.6)	20 (21.5)	7 (15.6)	13 (27.1)

Abbreviations: HRQoL: health-related quality of life; EQ5D: EuroQol-5D.
[a] P < .05. Baseline characteristics differ among control groups of completers vs non-completers of the 24-month visit.
[b] P < .05. Baseline characteristics differ among intervention groups of completers vs non-completers of the 24-month visit.

© 2024 Martínez-Ibáñez P et al. *JAMA Network Open.*

eTable 3. Home Blood Pressure Monitoring and Medication Changes at 24 Months

	Baseline	24 months	Mean change from baseline to 24 months	Mean difference between groups at 24 months
Home blood pressure monitoring, % (95% CI)				
Intervention	27.9 (19.8, 37.2)	93.7 (87.4, 97.4)	65.8 (56.3, 75.3)	53.0 (42.6, 63.3)
Control	33.3 (24.6, 43.1)	40.7 (31.4, 50.6)	7.4 (5.4, 20.2)	p < 0.001
Number of antihypertensive drugs, mean (95% CI)				
Intervention	1.8 (1.6, 2.0)	2.4 (2.2, 2.5)	0.6 (0.4, 0.7)	0.3 (0.02, 0.52)
Control	1.6 (1.4, 1.8)	2.1 (1.9, 2.3)	0.5 (0.3, 0.6)	p=0.04

© 2024 Martínez-Ibáñez P et al. *JAMA Network Open.*

eTable 4. Self-Titration of Antihypertensive Medication in the Intervention Group (n=111) During the Whole Study Period and the Extension Phase

	Patients with at least one treatment modification, No. (%)	Number of treatment modifications, Mean (95% CI)
Whole study period		
Dose increased	43 (38.7)	1.8 (1.4 to 2.2)
New medication added	61 (55.0)	1.9 (1.4 to 2.3)
Any self-titration	76 (68.5)[a]	2.7 (2.2 to 3.3)
Extension phase (from 12 to 24 month).		
Dose increased	11 (9.9)	1.6 (1.1 to 2.0)
New medication added	21 (18.9)	1.9 (1.1 to 2.7)
Any self-titration	31 (27.9)[a]	2. (1.5 to 2.9)

[a] At least one treatment modification through self-titration (either an increase in dose or an addition of a new medication)

© 2024 Martínez-Ibáñez P et al. *JAMA Network Open.*

eTable 5. Differences in Systolic and Diastolic Blood Pressure Among Study Groups at 24 Months, Crude And Adjusted: Sensitivity Analysis Including All Patients Attending the 12-Month Visit (n=312) Using Multiple Imputation[a]

	**Mean (95% CI)**					
	**Blood pressure**			**Reduction from baseline to 24 months**	**Reduction from 12 to 24 months**	**Difference between groups at 24 months**
	**Baseline**	**12 months**	**24 months**			
Systolic blood pressure						
Intervention	155.2 (153.1 to 157.2)	136.2 (134.0 to 138.4)	133.8 (131.0 to 136.7)	-21.3 (-24.4 to -18.2)	-2.4 (-5.4 to 0.6)	-3.7 (-7.7 to 0.4) p = 0.08
Control	155.2 (153.2 to 157.3)	139.2 (136.8 to 141.6)	137.5 (134.8 to 140.1)	-17.8 (-20.7 to -14.9)	-1.7 (-4.7 to 1.3)	
Diastolic blood pressure						
Intervention	89.7 (88.4 to 90.9)	80.9 (79.5 to 82.3)	80.6 (78.9 to 82.4)	-9.0 (-10.8 to -7.2)	-0.3 (-2.0 to 1.4)	-2.4 (-4.9 to 0.1) p = 0.06
Control	90.6 (89.3 to 91.9)	83.0 (81.5 to 84.6)	83.0 (81.3 to 84.8)	-7.6 (-9.4 to -5.7)	0.0 (-2.0 to 2.0)	
Systolic blood pressure, adjusted[b]						
Intervention			133.9 (133.1 to 134.6)[c]			-3.6 (-4.7 to -2.5) p < 0.001
Control			137.4 (136.7 to 138.2)[c]			
Diastolic blood pressure, adjusted[b]						
Intervention			80.5 (80.0 to 81.0)[c]			-2.5 (-3.2 to -1.7) p < 0.001
Control			83.0 (82.4 to 83.5)[c]			

[a] This analysis includes all patients attending the 12-month visit (n=312), missing BP measurements at 24 months were imputed using multiple-imputation analyses based on a “Markov chain Monte Carlo” (MCMC) simulation.
[b] Adjusted for sex, age, baseline systolic blood pressure, obesity, diabetes (fixed effects) and general practitioner (random effect).
[c] Mean prediction from the fitted model.

© 2024 Martínez-Ibáñez P et al. *JAMA Network Open.*

eTable 6. Differences in Systolic and Diastolic Blood Pressure Among Study Groups at 24 Months, Crude and Adjusted: Sensitivity Analysis Including All Patients Attending the 12-Month Visit (n=312) Using the Last Observation Carried Forward[a]

	**Mean (95% CI)**					
	**Blood pressure**			**Reduction from baseline to 24 months**	**Reduction from 12 to 24 months**	**Difference between groups at 24 months**
	**Baseline**	**12 months**	**24 months**			
Systolic blood pressure						
Intervention	155.2 (153.1 to 157.2)	136.2 (134.0 to 138.4)	134.7 (132.4 to 137.1)	-20.4 (-23.2 to-17.7)	-1.5 (-3.3 to 0.3)	-3.2 (-6.5 to 0.1)
Control	155.2 (153.2 to 157.3)	139.2 (136.8 to 141.6)	137.9 (135.6 to 140.2)	-17.3 (-19.9 to -14.8)	-1.3 (-3.4 to 0.9)	p = 0.058
Diastolic blood pressure						
Intervention	89.7 (88.4 to 90.9)	80.9 (79.5 to 82.3)	80.4 (79.0 to 81.9)	-9.2 (-10.7 to -7.7)	-0.5 (-1.6 to 0.6)	-2.3 (-4.3 to -0.3)
Control	90.6 (89.3 to 91.9)	83.0 (81.5 to 84.6)	82.7 (81.4 to 84.1)	-7.9 (-9.1 to -6.6)	-0.3 (-1.6 to 1.0)	p = 0.025
Systolic blood pressure, adjusted[b]						
Intervention			134.7 (133.9 to 135.4)[c]			-3.1 (-4.1 to -2.1)
Control			137.3 (136.4 to 138.3)[c]			p < 0.001
Diastolic blood pressure, adjusted[b]						
Intervention			80.2 (79.6 to 80.7)[c]			-2.5 (-3.2 to -1.7)
Control			82.7 (82.1 to 83.2)[c]			p < 0.001

[a] This analysis include all patients attending the 12-month visit (n=312), including patients with missing BP measurements at 24 months using the last observation carried forward for those whose last BP measurements were at 12 months
[b] Adjusted for sex, age, baseline systolic blood pressure, obesity, diabetes (fixed effects) and general practitioner (random effect).
[c] Mean prediction from the fitted model.

© 2024 Martínez-Ibáñez P et al. *JAMA Network Open.*

eTable 7. Number and Percentage of Patients Achieving Their Target BP at the Final Follow-Up Visit in the Intervention and Control Groups

	No. (%)		
	**Intervention**	**Control**	**Difference**
On target	71 (64.0)	58 (53.7)	*10.3 (*-2.7 to 23.2)
Off target	40 (36.0)	50 (46.3)	p = 0.06

© 2024 Martínez-Ibáñez P et al. *JAMA Network Open.*

eTable 8. Adverse Events During the Extension Follow-Up Period

	No. (%)	
	Intervention (n=111)	Control (n=108)
**Adverse events potentially related to hypertension, antihypertensive treatment and/or intervention**		
Swelling of legs and/or ankles	1 (0.9)	0 (0.0)
Diarrhea	1 (0.9)	0 (0.0)
Gait disturbance	0 (0.0)	1 (0.9)
Nephrolithiasis	1 (0.9)	0 (0.0)
Muscular weakness	1 (0.9)	0 (0.0)
Hacking cough	0 (0.0)	1 (0.9)
Stroke	0 (0.0)	3 (2.7)
Dizziness	0 (0.0)	1 (0.9)
**Serious adverse events**[a]		
Breast cancer	2 (1.3)	1 (0.6)
Diverticular bleeding[b]	0 (0.0)	1 (0.9)
Acute prostatitis[b]	1 (0.9)	0 (0.0)
Stroke[c]	0 (0.0)	3 (2.7)
Parkinson's disease[b]	1 (0.9)	0 (0.0)
Spinal meningioma	1 (0.9)	0 (0.0)
Dacryocystitis[b]	0 (0.0)	1 (0.9)
Urinary incontinence[b]	0 (0.0)	1 (0.9)

[a]Defined as any clinical event requiring hospitalization, endangering the patient’s life, or having an otherwise substantial impact on the patient’s health, as determined by the researcher;
[b]Adverse event that required hospitalization; ARBs: angiotensin-receptor blockers.
[c] This adverse event is also included under the category “Adverse events potentially related to hypertension, antihypertensive treatment and/or intervention”.

© 2024 Martínez-Ibáñez P et al. *JAMA Network Open.*

eTable 9. Behavioural Risks and Health-Related Quality of Life at 12 Months and at the Final Follow-Up Visit

	% (95% CI)			
	**12 months**	**24 months**	**Mean reduction from 12 months to 24 months**	**Mean difference between groups at 24 months**
Smoking				
Intervention	16.2 (9.4 to 23.1)	16.2 (9.4 to 23.1)	0.0 (-0.1 to 0.1)	-4.2 (-14.4 to 6.1)
Control	20.4 (12.8 to 28.0)	23.2 (15.2 to 31.1)	-2.8 (-13.8 to 8.2)	p = 0.43
Sedentarism				
Intervention	34.2 (25.4 to 43.1)	34.2 (25.4 to 43.1)	0.0 (-12.5 to 12.5)	-5.6 (-18.4 to 7.2)
Control	37.0 (27.9 to 46.1)	39.8 (30.6 to 49.0)	27.8 (-10.2 to 15.7)	p = 0.39
Obesity				
Intervention	38.7 (29.7 to 47.8)	40.5 (31.4 to 49.7)	1.8 (-11.1 to 14.7)	4.8 (-17.9 to 8.3)
Control	45.4 (36.0 to 54.8)	45.4 (36.0 to 54.8)	0.0 (-13.3 to 13.3)	p = 0.47
Health-related quality of life (EQ5D), mean (95% CI)				
Intervention	0.86 (0.82 to 0.90)	0.85 (0.82 to 0.89)	-0.01 (-0.04 to 0.03)	0.00 (-0.05 to 0.05)
Control	0.86 (0.82 to 0.89)	0.86 (0.82 to 0.89)	0.00 (-0.04 to 0.04)	p = 0.90

© 2024 Martínez-Ibáñez P et al. *JAMA Network Open.*

eTable 10. Health Care Services Utilization During the Extension Follow-Up Period

	No. (%)			
	**Total (n=219)**	**Intervention (n=111)**	**Control (n=108)**	**p value**
Visits to the health centre with appointment				
0-2	135 (61.6)	72 (64.9)	63 (58.3)	0.15
3-6	65 (29.7)	27 (24.3)	38 (35.2)	
7 or more	19 (8.7)	12 (10.8)	7 (6.5)	
Mean (SD)	2.74 (2.4)	2.70 (2.5)	2.77 (2.3)	0.84
Visits to the health centre without appointment				
None	199 (90.9)	106 (95.5)	93 (86.1)	0.05
1	13 (5.9)	3 (2.7)	10 (9.3)	
2 or more	7 (3.2)	2 (1.8)	5 (4.6)	
Mean (SD)	0.12 (0.4)	0.06 (0.3)	0.19 (0.5)	0.03
Calls to the healthcare centre				
None	214 (97.7)	108 (97.3)	106 (98.15)	0.18
1 or more	5 (2.3)	3 (2.7)	2 (1.85)	
Home visits				
None	218 (99.5)	110 (99.1)	108 (100.0)	0.98
1	1 (0.5)	1 (0.9)	0.0 (0.0)	

© 2024 Martínez-Ibáñez P et al. *JAMA Network Open.*

eFigure. Mean SBP and DBP in Follow-Up Visits at Baseline, 6, 12 Months and the Final Follow-Up Visit for the Intervention (n=111) and Control Groups (n=108)

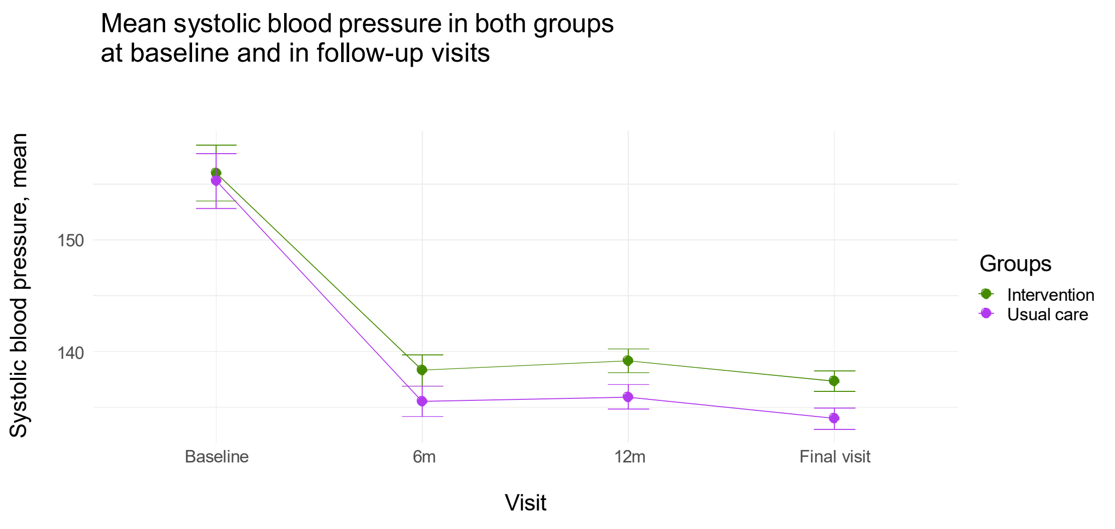

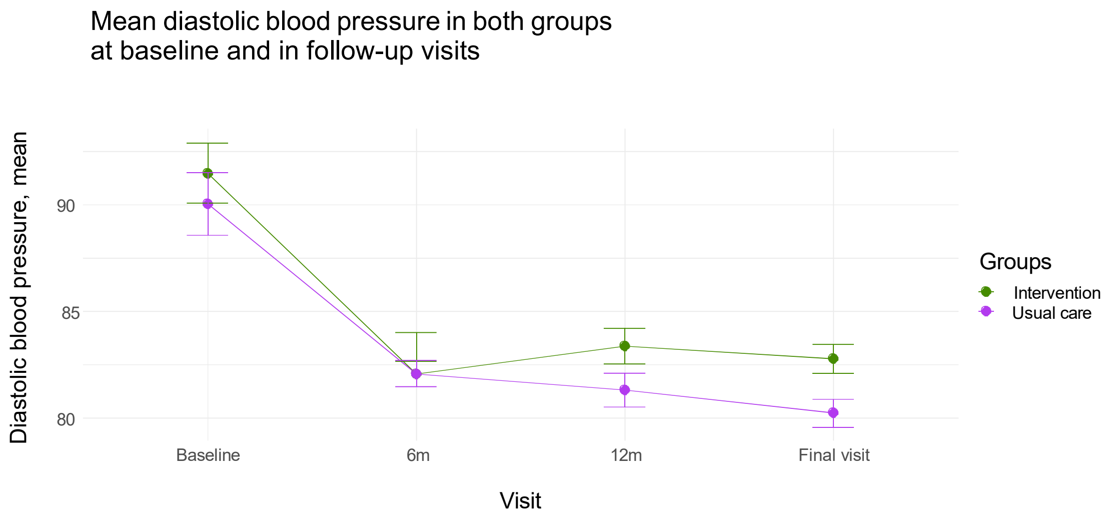

© 2024 Martínez-Ibáñez P et al. *JAMA Network Open.*
