# Supplementary material for: Long-Term Effect of Home Blood Pressure Self-Monitoring Plus Medication Self-Titration for Patients With Hypertension: A Secondary Analysis of the ADAMPA Randomized Clinical Trial
Source: JAMA Netw Open. 2024 May 10;7(5):e2410063. doi: 10.1001/jamanetworkopen.2024.10063 (PMC11087839; doi:10.1001/jamanetworkopen.2024.10063)
Supplement: Supplement 3. — Nonauthor Collaborators [file jamanetwopen-e2410063-s003.pdf]

Supplemental Online Content: Nonauthor Collaborators
*First name, last name, and suffix (if applicable) are required and will appear in PubMed.

*Group Name(s): ADAMPA Research Group							
***First Name and Middle Initial(s)**	***Last Name**	***Suffix (eg, Jr, III)**	Academic Degrees	Institution	Location (city, state/province, country)	Role or Contribution, eg, chair, principal investigator	Group (if more than 1 Group listed in the byline) and/or Subgroup (eg, Steering Committee)
Joaquín	Abad Carrasco			INCLIVA		investigator	
Maria Virginia	Agudo Escagüés			INCLIVA		investigator	
Jorge	Navarro-Perez			INCLIVA		investigator	
Rosa Maria	Bartual Penella			INCLIVA		investigator	
Rosa	Carrión Villanueva			INCLIVA		investigator	
Ana	Costa Alcaraz			INCLIVA		investigator	
Isabel	Cristófol López			INCLIVA		investigator	
Rosario	González Candelas			INCLIVA		investigator	
Ricardo	González Espadas			INCLIVA		investigator	
Luis	González Luján			INCLIVA		investigator	
Victoria	Gosalbes			INCLIVA		investigator	
Enrique	Guinot Martínez			INCLIVA		investigator	
Emilio Luis	López Torres			INCLIVA		investigator	
Silvia	Molla LLosa			INCLIVA		investigator	
Víctor	Moreno Comins			INCLIVA		investigator	
Miriam	Moreno Prat			INCLIVA		investigator	
Mª José	Puchades Company			INCLIVA		investigator	
Ángela	Ramos García			INCLIVA		investigator	
Paloma	Ramos Ruiz			INCLIVA		investigator	
Pilar	Roca Navarro			INCLIVA		investigator	
Rosa	Saiz Rodriguez			INCLIVA		investigator	
Julia Lorena	Salanova Chilet			INCLIVA		investigator	
Ana	Tchang Sanchez			INCLIVA		investigator	
Francisca	Torres Asensi			INCLIVA		investigator	
Ruth	Uribes Fillol			INCLIVA		investigator	
Cristina	Valle García			INCLIVA		investigator	
Macarena	Villar Ruiz			INCLIVA		investigator	
Marta	Alcocer Escribano			INCLIVA		investigator	
Laura	Almudever Campo			INCLIVA		investigator	

Supplemental Online Content: Nonauthor Collaborators
*First name, last name, and suffix (if applicable) are required and will appear in PubMed.

***First Name and Middle Initial(s)**	***Last Name**	***Suffix (eg, Jr, III)**	Academic Degrees	Institution	Location (city, state/province, country)	Role or Contribution, eg, chair, principal investigator	Group (if more than 1 Group listed in the byline) and/or Subgroup (eg, Steering Committee)
Lorena	Cruz Bautista			INCLIVA		investigator	
Mª Begoña	Fuertes Fernandez			INCLIVA		investigator	
Victor	García Olivencia			INCLIVA		investigator	
Carmen	Molla Orts			INCLIVA		investigator	
María José	Muñoz Sanchíz			INCLIVA		investigator	
Francisca	Osuna Sabariego			INCLIVA		investigator	
Emilia	Ramón Carretero			INCLIVA		investigator	
Pilar	Roca Roda			INCLIVA		investigator	
Esther	Rodriguez García			INCLIVA		investigator	
Maria Rosa	Serrada Iranzo			INCLIVA		investigator	
Eva	Sierra García			INCLIVA		investigator	
Adina A	Iftimi			Valencia University		investigator	
Andreu	Ferrero-Gregori			INCLIVA		investigator	
