# Supplementary material for: Long-Term Effect of Home Blood Pressure Self-Monitoring Plus Medication Self-Titration for Patients With Hypertension: A Secondary Analysis of the ADAMPA Randomized Clinical Trial
Source: JAMA Netw Open. 2024 May 10;7(5):e2410063. doi: 10.1001/jamanetworkopen.2024.10063 (PMC11087839; doi:10.1001/jamanetworkopen.2024.10063)
Supplement: Supplement 4. — Data Sharing Statement [file jamanetwopen-e2410063-s004.pdf]

Data Sharing Statement

Martínez-Ibáñez. Long-Term Effect of Home Blood Pressure Self-Monitoring Plus Medication Self-Titration for Patients With Hypertension. *JAMA Netw Open*. Published May 10, 2024. doi:10.1001/jamanetworkopen.2024.10063

Data
Data available: Yes
Data types: Deidentified participant data
How to access data: The datasets used and/or analysed during the current study are available from the corresponding author on reasonable request.
When available: With publication

Supporting Documents
Document types: None

Additional Information
Who can access the data: Anyone requesting the data.
Types of analyses: For a specified purpose.
Mechanisms of data availability: Without investigator support.
Any additional restrictions: Deidentified data may be available to others upon request to the corresponding author pending approval by the IRB/Research Ethics Committee
